# PAQR8 promotes breast cancer recurrence and confers resistance to multiple therapies

**DOI:** 10.1186/s13058-022-01559-3

**Published:** 2023-01-03

**Authors:** Saisai Chen, Matt R. Paul, Christopher J. Sterner, George K. Belka, Dezhen Wang, Peining Xu, Amulya Sreekumar, Tien-chi Pan, Dhruv K. Pant, Igor Makhlin, Angela DeMichele, Clementina Mesaros, Lewis A. Chodosh

**Affiliations:** 1 Abramson Family Cancer Research Institute, Perelman School of Medicine, University of Pennsylvania, Room 614 BRB II/III, 421 Curie Boulevard, Philadelphia, PA 19104-6160 USA; 2 2-PREVENT Translational Center of Excellence, Perelman School of Medicine, University of Pennsylvania, Philadelphia, PA 19104 USA; 3 Department of Cancer Biology, Perelman School of Medicine, University of Pennsylvania, Philadelphia, PA 19104 USA; 4 Department of Medicine, Perelman School of Medicine, University of Pennsylvania, Philadelphia, PA 19104 USA; 5 Systems Pharmacology and Translational Therapeutics, Perelman School of Medicine, University of Pennsylvania, Philadelphia, PA 19104 USA

**Keywords:** Breast cancer, Tumor recurrence, Treatment resistance, Endocrine therapy, Translational research

## Abstract

**Background:**

Breast cancer mortality is principally due to recurrent disease that becomes resistant to therapy. We recently identified copy number (CN) gain of the putative membrane progesterone receptor *PAQR8* as one of four focal CN alterations that preferentially occurred in recurrent metastatic tumors compared to primary tumors in breast cancer patients. Whether PAQR8 plays a functional role in cancer is unknown. Notably, *PAQR8* CN gain in recurrent tumors was mutually exclusive with activating *ESR1* mutations in patients treated with anti-estrogen therapies and occurred in > 50% of both patients treated with anti-estrogen therapies and those treated with chemotherapy or anti-Her2 agents.

**Methods:**

We used orthotopic mouse models to determine whether *PAQR8* overexpression or deletion alters breast cancer dormancy or recurrence following therapy. In vitro studies, including assays for colony formation, cell viability, and relative cell fitness, were employed to identify effects of PAQR8 in the context of therapy. Cell survival and proliferation were quantified by immunofluorescence staining for markers of apoptosis and proliferation. Sphingolipids were quantified by liquid chromatography-high resolution mass spectrometry.

**Results:**

We show that PAQR8 is necessary and sufficient for efficient mammary tumor recurrence in mice, spontaneously upregulated and CN gained in recurrent tumors that arise following therapy in multiple mouse models, and associated with poor survival following recurrence as well as poor overall survival in breast cancer patients. PAQR8 promoted resistance to therapy by enhancing tumor cell survival following estrogen receptor pathway inhibition by fulvestrant or estrogen deprivation, Her2 pathway blockade by lapatinib or Her2 downregulation, and treatment with chemotherapeutic agents. Pro-survival effects of PAQR8 were mediated by a G_i_ protein-dependent reduction in cAMP levels, did not require progesterone, and involved a PAQR8-dependent decrease in ceramide levels and increase in sphingosine-1-phosphate levels, suggesting that PAQR8 may possess ceramidase activity.

**Conclusions:**

Our data provide in vivo evidence that PAQR8 plays a functional role in cancer, implicate PAQR8, cAMP, and ceramide metabolism in breast cancer recurrence, and identify a novel mechanism that may commonly contribute to the acquisition of treatment resistance in breast cancer patients.

**Supplementary Information:**

The online version contains supplementary material available at 10.1186/s13058-022-01559-3.

## Background

Breast cancer is the most frequently diagnosed cancer and leading cause of cancer-related death among women worldwide [1]. Most deaths from breast cancer are due to treatment-refractory recurrences that occur months or years after definitive treatment of the primary tumor [2]. Consequently, a better understanding of the mechanisms underlying disease recurrence, particularly therapy resistance, is essential for improving patient outcomes.

Systemic therapies for breast cancer, including anti-estrogen therapies, anti-Her2 therapies, and chemotherapy, are selected based upon tumor subtype (i.e., estrogen receptor and Her2 status), tumor stage, lymph node status, and other biological features relevant to risk stratification. Despite advances in treatment, however, up to 30% of patients eventually develop recurrent disease, which is typically incurable due to therapy resistance that develops to each of these classes of agents [2]. Thus, resistance to therapy is a core feature of breast cancer progression that is disproportionately responsible for mortality.

To address this critical gap in knowledge, Paul et al. [3] recently performed whole exome and shallow whole genome sequencing of paired primary and recurrent breast cancers in patients in the METAMORPH study who had undergone anti-estrogen therapy, chemotherapy, and/or anti-HER2 therapy prior to tumor recurrence. Across the genome, only four regions with copy number (CN) alterations were determined to be preferentially enriched in recurrent, compared to primary, tumors, including preferential CN loss of the tumor suppressors *STK11/LKB1* and *CDKN2A*, and preferential CN gain of *PTK6*. A fourth focal 78 kbp region on chromosome 6p12.2 exhibited preferential CN gain in recurrent tumors and contained the entire coding sequence of only a single gene, *PAQR8*. To date, an in vivo role for PAQR8 in cancer has not been reported, and little is known about its function or mechanism of action.

Given that *STK11/LKB1*, *CDKN2A*, and *PTK6* each have well-described roles in cancer progression [4–6], we hypothesized that *PAQR8* might play a role in breast cancer recurrence. A clue to its potential role came from the finding that, in patients treated with anti-estrogen therapy, *PAQR8* CN gain in recurrent tumors was mutually exclusive with activating point mutations in *ESR1*, as well as mutations in *PGR* [3]. *ESR1* mutations occur in up to 20% of recurrent breast cancers arising in patients treated with anti-estrogen therapies and occur almost exclusively in patients treated with aromatase inhibitors [7, 8]. Since activating *ESR1* mutations confer resistance to anti-estrogen therapy, this mutually exclusive pattern suggested that PAQR8 might play a redundant role in this process, whether by activating a downstream mediator of the estrogen receptor (ER) itself or an alternative resistance pathway.

Notably, *PAQR8* CN gain in recurrent breast cancers was not restricted to patients who received anti-estrogen therapy as it was found with equal frequency among patients treated with other therapies, including chemotherapy and agents targeting Her2. These findings suggested the intriguing possibility that PAQR8 might promote a more generalized form of therapy resistance and, in that way, contribute to tumor recurrence.

PAQR8, also known as mPRβ, is a member of the membrane progesterone receptor (mPR) subclass of the progestin and adipoQ receptor (PAQR) family [9, 10]. PAQR8 expression has been detected in a variety of cell types, including normal and malignant breast, ovarian, and myometrial cells [11–13]. Its function is poorly understood, and there have been conflicting reports regarding PAQR8 structure, function, and ability to bind and respond to progesterone [11, 14–16].

Membrane progesterone receptors have been reported to mediate rapid effects of progesterone signaling. Although residues that disrupt progesterone binding when mutated have recently been reported for the related mPR family member, PAQR7 [17], a progesterone binding site for PAQR8 has not been identified. While siRNA knockdown of PAQR8 in cells has been reported to reduce membrane progesterone binding, it did not affect progesterone-mediated downstream functions [11, 18]. Further, in a yeast model in which human PAQR8 was expressed, progesterone was not required for activity [19]. Thus, whether progesterone plays a role in PAQR8 function remains uncertain.

Based on computational predictions, PAQR8 has been proposed to be a 7-transmembrane protein with an extracellular N-terminus and intracellular C-terminus resembling GPCRs [9, 10]. Consistent with a GPCR-like function, some evidence suggests that effects of PAQR8 and other mPRs may be mediated by coupling to an inhibitory G (G_i_) protein [11, 20]. However, there is as yet no direct evidence that PAQR8 acts to decrease intracellular cAMP levels in a manner that is sensitive to pertussis toxin (PTX), a potent inhibitor of GPCRs coupled to G_i_ proteins [21].

It has also been proposed that PAQR8 might potentially function as an alkaline ceramidase based on the presence of three motifs conserved across the entire PAQR family that resemble motifs conserved within the alkaline ceramidase family with respect to both sequence and location [22, 23]. Indeed, the yeast PAQR protein Izh2p, as well as human adiponectin receptors PAQR1 and PAQR2, have been reported to possess intrinsic ceramidase activity [24, 25]. However, no evidence to date has shown that PAQR8 can alter levels of ceramides or other sphingolipids in eukaryotic cells.

In this study, we examined the role of PAQR8 in breast cancer recurrence and resistance to therapy. Our data indicate that PAQR8 plays a functional role in cancer progression by demonstrating that PAQR8 is both necessary and sufficient to promote efficient breast cancer recurrence and that PAQR8 confers resistance to each of the classes of therapeutic agents commonly used in breast cancer patients by enhancing cell survival, and does so independently of progesterone. We further show that the pro-survival effects of PAQR8 in cancer cells are dependent upon a G_i_ protein-mediated reduction in intracellular cAMP levels and demonstrate that PAQR8 alters the balance of ceramides and sphingolipids in a manner predicted to promote cell survival. Consistent with a clinically relevant role in breast cancer, we found that PAQR8 is spontaneously upregulated and CN gained in post-therapy recurrent tumors from multiple mouse models and is associated with breast cancer progression and poor survival in patients. Together, our data support a model in which PAQR8 promotes breast cancer recurrence by facilitating resistance to multiple antineoplastic therapies in a manner that alters ceramide levels and requires coupling to a G_i_ protein.

## Methods

### Genomic data

Within the METAMORPH cohort [3, 26], the association between mutations in *ESR1*, mutations in *PGR*, or gain of *PAQR8* with survival following clinical progression after recurrence was assessed using Cox proportional hazards regression. Variant and copy number calling methodologies were previously described [3].

Within the TCGA BRCA cohort [8], survival data and copy number calls from GISTIC2 were obtained from the NIH Genomic Data Commons (GDC) portal [27]. + 1 and + 2 GISTIC2 calls were considered to represent low- and high-level gain, respectively. Associations with *PAQR8* gain were assessed using Cox proportional hazards regression.

For primary and recurrent tumors arising in GEM models, CN change calls were identified using shallow whole genome sequencing (75 bp single-end reads, 30.1 million reads on average, 0.9X coverage), alignment using BWA [28], CN signal quantification using QDNAseq [29], and CN normalization using ACE [30]. Low-level CN gains (≥ 2.12) were defined as CN calls that were detected above the threshold that contained > 99% of copy number calls in normal tissue samples. The threshold for high-level CN gain (≥ 2.9) was defined based on the ability to encompass the majority of the CN distribution peak at CN = 3. Low-level gains were considered to represent subclonal CN events, and high-level gains were considered to represent either clonal + 1 CN events or subclonal >  + 1 CN events. Frequencies of CN gain were compared between primary and recurrent tumors using one-sided Fisher’s exact test. mRNA expression was assayed following TruSeq RNA library preparation and sequencing (100 bp paired-end reads, 30 M reads on average), followed by STAR alignment [31], read quantification by featureCounts [32], and read normalization using DESeq2 [33]. Normalized read counts for *Paqr8* were compared using Wilcoxon rank-sum test.

### Tissue culture

Inducible Her2-dependent primary mouse tumor cells were cultured as described [34]. MCF7, BT474-M1 cells, and SUM159 cells were purchased from ATCC and cultured as recommended. Charcoal-stripped serum was purchased from Sigma-Aldrich (#F6765, lot 18L289).

Viable cell numbers were measured using a Vi-CELL cell counter (BD Biosciences). For colony formation assays, cells were plated in 6-well plates in complete growth medium and then shifted to the specified test medium 24 h later. Colonies were fixed and stained with crystal violet for visualization and quantified manually.

### Drugs

Lapatinib (#S2111), doxorubicin (#S1208), and docetaxel (#S1148) were purchased from Selleck Chemicals. Fulvestrant (#I4409) and pertussis toxin (#P7208) were purchased from Sigma-Aldrich. Forskolin was purchased from Abcam (#ab120058).

### Plasmids and lentivirus production

Plasmids pUltra (#24129) and pUltra-hot (#24130) were purchased from Addgene. The eGFP and mCherry alleles were replaced with H2B-eGFP and H2B-mCherry from Addgene plasmids #11680 and #20972, respectively. Human and mouse *PAQR8* cDNA sequences were synthesized by Integrated DNA Technologies, sequence-verified, and cloned into the above-modified pUltra plasmid. Sense and anti-sense oligos for each sgRNA were ligated into BsmB1-digested LRG2.1 vector (Addgene #108098) or LRmCherry2.1 vector (Addgene #108099). Lentivirus was produced by transfecting HEK293T cells with polyethylenimine (Polysciences #23966), pMD2.G (Addgene #12259), psPAX2 (Addgene #12260), and the plasmid of interest.

### Mouse experiments

Animal care and experiments were performed in accordance with the guidelines of the University of Pennsylvania IACUC. Recurrence assays were performed as described [34, 35]. Briefly, 1 × 10^6^ cells were injected into the inguinal mammary fat pads of female *nu/nu* mice. Mice were maintained on 2 mg/ml doxycycline in drinking water until primary tumors reached 5 × 5 mm. Mice were palpated 2–3 times weekly for tumor recurrence. To form MCF7 tumors, 2.5 × 10^6^ MCF7 cells were injected into the inguinal mammary fat pads of female *NSG* mice. Mice were palpated twice weekly for tumor formation and sacrificed when tumors reached 8 × 8 mm. All mice were injected with 50 mg/kg EdU (i.p.) 2 h prior to sacrifice.

### Droplet digital PCR

Genomic DNA isolation from cells was performed using the QIAamp DNA mini kit (Qiagen #51306) following manufacturer’s instructions. Genomic DNA isolation from tissue was performed using Zymo Quick-DNA Midiprep Plus kit (Zymo Research #D4075) following manufacturer’s instructions. Probes were all purchased from Bio-Rad, including eGFP (#dCNS372378948), mCherry (#dCNS507694046), and ApoB (#dMmuCNS407594696). Droplets were generated and read using the QX200 AutoDG Droplet Digital PCR System (Bio-Rad). PCR was performed using the C1000 Touch Thermal Cycler (Bio-Rad).

### Immunofluorescence

Mammary tumors were fixed in 4% PFA, dehydrated, and embedded in paraffin blocks. Paraffin tissue sections 8 µm thick were prepared using a standard xylene-based dewaxing procedure [36]. Sections were subjected to antigen retrieval in Buffer A or B (Electron Microscopy Sciences) using a 2100 Retriever (Diagnostic Technology). Cells grown on coverslips were fixed in 4% PFA and permeabilized in 0.5% Triton X-100. Blocking was performed in 3% BSA and 5% normal goat serum for 1 h before overnight incubation at 4 °C with primary antibodies. Samples were incubated with secondary antibodies for 1 h at room temperature followed by Hoechst staining for nuclei. Slides were visualized on a DM 5000B Automated Upright Microscope (Leica), and images were captured with a DFC350 FX monochrome digital camera (Leica). Images were quantified using QuPath-0.3.0 software [37].

### Antibodies

Primary antibodies and dilutions included: chicken anti-GFP (Abcam #ab13970; 1:1000), mouse anti-GFP (Living Colors #JL-8; 1:250), rabbit anti-cleaved caspase-3 (Cell Signaling #9664; 1:250), rat anti-Ki67 (eBioscience #14-5698; 1:100), rat anti-HA (Roche #3F10; 1:1000), and anti-β-tubulin (BioGenex #MU122-UC; 1:5000). EdU detection: In Vivo EdU Click Kit 647 (Sigma-Aldrich #BCK647-IV-IM-S), in vitro Click-iT Plus EdU Cell Proliferation Kit for Imaging Alexa Fluor 647 dye (Thermo Fisher Scientific #C10640). Secondary Alexa-conjugated antibodies (Invitrogen; 1:1000) included: goat anti-chicken IgG Alexa-488 (#A11039), goat anti-mouse IgG2A Alexa-488 (#A21131), goat anti-rabbit IgG Alexa-594 (#A11012), and goat anti-rat IgG Alexa-568 (#A11077). Secondary IRDye-conjugated antibodies (LiCor; 1:10,000) included: 680LT anti-mouse IgG1 (#926-68050) and 800CW anti-rat IgG (#926-32219).

### cAMP

Intracellular cAMP levels were measured using a fluorometric competitive ELISA assay kit (Abcam #ab138880) following manufacturer’s instructions.

### Liquid chromatography-high resolution mass spectrometry

Sphingolipid extraction was performed as described [38] after adding 1 mL of 80% methanol to each plate to inactivate enzymes. Each experimental condition had five technical replicates, with the sixth used for protein quantification. Sphingolipids levels were normalized to protein content.

### Statistics

Two-tailed Student’s *t* tests were used to assess differences between groups, with the exception of the Mann–Whitney *U* test that was employed for data that were not normally distributed, as determined by the Shapiro–Wilk test. Survival curves were generated using the Kaplan–Meier method, with P values and hazard ratios calculated by the Mantel–Haenszel method and log-rank test for trend. *P* < 0.05 was considered statistically significant. In vitro analyses are representative of at least three independent experiments.

## Results

### PAQR8 gain is associated with breast cancer progression

**Fig. 1:**
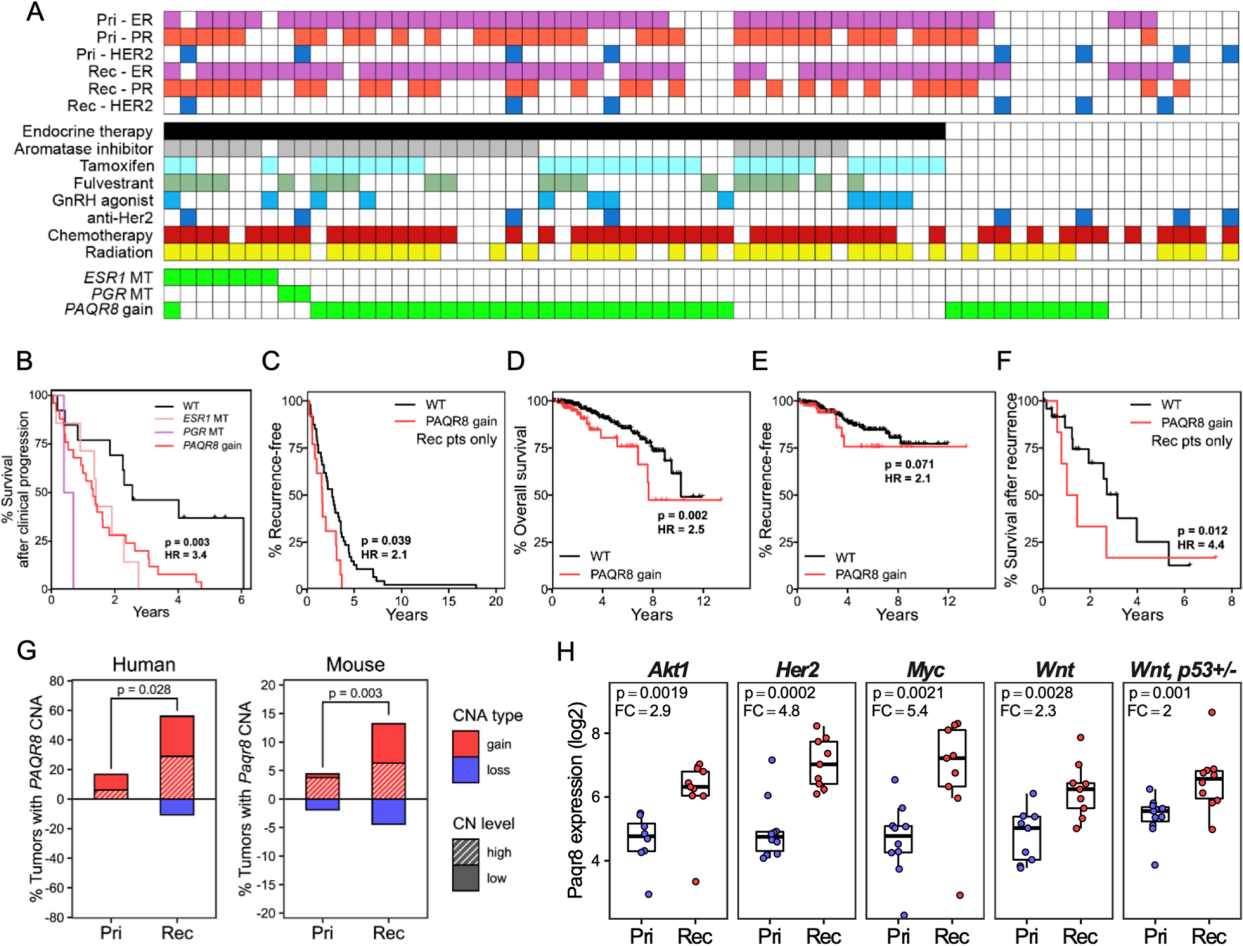
*PAQR8* gain in human and mouse tumors is associated with recurrence and disease progression. **A** Patients in the METAMORPH cohort, depicting receptor status in primary (Pri) and recurrent (Rec) tumors, treatments received, and mutation or copy number status. **B** Cox proportional hazards regression analysis of survival after clinical progression following recurrence for patients whose recurrent tumors had activating mutations in *ESR1* (*n* = 7), mutations in *PGR* (*n* = 2), gain of *PAQR8* (*n* = 26), or patients with tumors lacking these three alterations (WT, *n* = 13). **C**–**F** Cox proportional hazards regression analysis of different survival outcomes with respect to *PAQR8* gain in primary tumors within the TCGA breast cancer dataset. **C** Time to recurrence in patients who experienced recurrence across all subtypes controlling for HR/HER2 status (*n* = 49 WT, 11 *PAQR8* gain). Overall survival (*n* = 378 WT, 72 *PAQR8* gain) (**D**), recurrence-free survival (*n* = 354 WT, 66 *PAQR8* gain) (**E**), and survival after recurrence (*n* = 28 WT, 7 *PAQR8* gain) (**F**) in HR +/HER2- patients. **G** Frequencies of CN gain (red) and CN loss (blue) of *PAQR8/Paqr8* in primary and recurrent tumors in humans (left; *n* = 28 Pri, 66 Rec) and mice (right; *n* = 159 Pri, 169 Rec). Low-level CNAs (gain and loss) and high-level CNAs (amplification and deletion) are shown as hashed and solid bars, respectively. P values indicate one-sided Fisher’s exact tests. **H** *Paqr8* RNA expression in primary (*n* = 48) and recurrent (*n* = 46) tumors across five different GEM models. P values indicate one-sided Wilcoxon rank-sum tests. Median fold-change (FC)

Based on our findings in breast cancer patients in the METAMORPH study that *PAQR8* undergoes preferential CN gain in recurrent tumors, and that *PAQR8* CN gain is mutually exclusive with activating mutations in *ESR1* (Fig. 1A) [3], we sought to determine whether *PAQR8* gain was associated with clinical progression in this patient cohort [3]. Indeed, we found that patients treated with anti-endocrine therapies whose recurrent tumors harbored *PAQR8* gain, *ESR1* mutations, or *PGR* mutations had significantly poorer survival following clinical progression (*p* = 0.003, HR = 3.4) (Fig. 1B). Notably, survival outcomes for patients whose recurrent tumors harbored *PAQR8* gain were comparable to those with *ESR1* activating mutations (*p* = 0.6) (Fig. 1B).

Consistent with the above findings, *PAQR8* gain in primary breast cancers in The Cancer Genome Atlas (TCGA) was associated with shorter time to recurrence among those patients who recurred across all subtypes (*p* = 0.039, HR = 2.1) (Fig. 1C). Furthermore, *PAQR8* gain in primary tumors was associated with poor overall survival (*p* = 0.002, HR = 2.5), poor recurrence-free survival (*p* = 0.071, HR = 2.1), and poor survival following recurrence (*p* = 0.012, HR = 4.4) among HR+ /HER2– patients (Fig. 1D–F). These findings suggest that *PAQR8* CN gain may contribute to tumor recurrence, as well as tumor progression after recurrence, across different breast cancer subtypes.

### PAQR8 is upregulated and undergoes CN gain in recurrent tumors across multiple mouse models

To test the hypothesis that *PAQR8* CN gain promotes breast cancer recurrence, we employed bitransgenic doxycycline-inducible genetically engineered mouse (GEM) models containing transgenes for both *MMTV-rtTA* and *TetO-*driven oncogenes that permit the doxycycline-inducible expression of oncogenes of interest in a mammary epithelial-specific manner [35, 39–42]. These models faithfully recapitulate key elements of breast cancer progression, including primary tumor formation driven by the doxycycline-dependent expression of oncogenes important in human breast cancer, rapid tumor regression following oncogene downregulation as a manifestation of oncogene addiction, cellular dormancy in residual tumor cells that survive oncogene downregulation, and spontaneous tumor recurrence following a variable latency period [35, 39–42]. Dormant residual tumor cells isolated from these GEM models express a genetic signature that is strongly associated with recurrence-free survival in breast cancer patients [43], and studies using these GEM models have provided insights into pathways associated with clinical relapse [34, 36, 44, 45].

We first analyzed *Paqr8* CN and RNA levels in primary and recurrent tumors arising in five different GEM models in which primary tumorigenesis is driven by *Akt* (*MMTV-rtTA;TetO-Akt1*), *Her2* (*MMTV-rtTA;TetO-Her2*), *Myc* (*MMTV-rtTA;TetO-Myc*), *Wnt1* (*MMTV-rtTA;TetO-Wnt1*), or *Wnt1* in a *p53* ± background (*MMTV-rtTA;TetO-Wnt1;Trp53*^+*/−*^) [35, 39–42, 46, 47]. When compared to our findings in breast cancer patients [3], we found a striking similarity in the pattern of preferential enrichment for *Paqr8* CN gain in recurrent, compared to primary, tumors across these GEM models (Fig. 1G). In addition, we found that *Paqr8* mRNA levels were markedly upregulated in recurrent compared to primary tumors across all five GEM models (Fig. 1H).

Since recurrent tumors from GEM models arise in the setting of oncogene downregulation, this process is conceptually similar to the development of resistance to targeted therapies. Indeed, pharmacological inhibition of Her2 has been shown to yield similar effects to those observed for genetic downregulation of Her2 [48]. Thus, our finding that *Paqr8* undergoes preferential CN gain and is upregulated in recurrent tumors across multiple GEM models, recapitulating the preferential CN gain of *PAQR8* in recurrent breast cancers observed in patients, further suggests the hypothesis that Paqr8 promotes tumor recurrence and resistance to therapy.

### Paqr8 is both necessary and sufficient to promote efficient breast cancer recurrence

Given the parallel pattern of enrichment for *PAQR8* CN gain in recurrent tumors in both humans and mice, along with the observed upregulation of *Paqr8* during tumor recurrence in GEM models, we asked whether PAQR8 promotes breast cancer recurrence. To do this, we engineered Her2-dependent primary tumor cells [36, 43–45] derived from the *MMTV-rtTA;TetO-Her2* inducible GEM model mice [35] to either overexpress *Paqr8* or delete *Paqr8* using CRISPR-cas9 (Additional file 1, 11: Fig. S1, S11).

**Fig. 2:**
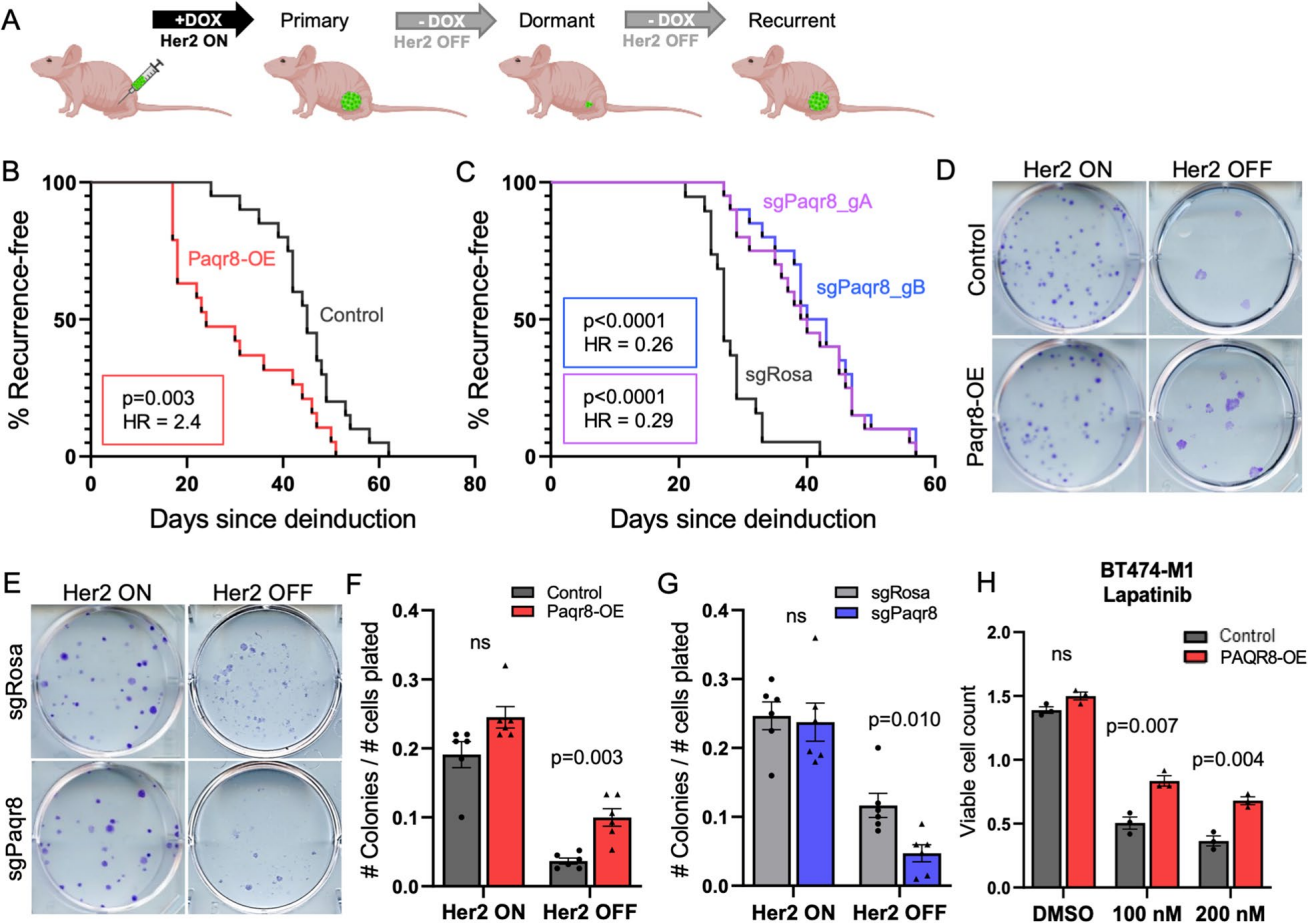
Paqr8 is necessary and sufficient for efficient tumor recurrence and colony formation following Her2 downregulation. **A** Schematic showing recurrence-free survival assays following orthotopic injection of Her2-dependent primary mouse tumor cells. Kaplan–Meier curves depicting rate of recurrence following Her2 downregulation comparing (**B**) Her2-dependent primary tumor cells overexpressing Paqr8 (*Paqr8-OE*) and controls, or (**C**) Her2-dependent primary tumor cells deleted for *Paqr8* using CRISPR-cas9 and two independent guide RNAs (*sgPaqr8*_gA, *sgPaqr8*_gB) and controls (*sgRosa*). Colony formation assays performed in medium containing 10% serum with doxycycline (Her2 ON) or in medium containing 1% serum without doxycycline (Her2 OFF), comparing (**D**) Her2-dependent primary tumor cells overexpressing Paqr8 and controls, or (**E**) cells deleted for *Paqr8* and *sgRosa* controls. **F**, **G** Bar graphs quantifying the proportion of plated cells that formed colonies under Her2 ON and OFF conditions. **H** Viable cell counts for BT474-M1 cells in medium containing lapatinib or vehicle control (DMSO)

Paqr8-overexpressing (*Paqr8-OE*) or control tumor cells were orthotopically injected into athymic nude (*nu/nu*) mice maintained on doxycycline. Following primary tumor formation, doxycycline was removed from drinking water, resulting in Her2 downregulation and tumor regression to a non-palpable state (Fig. 2A). Primary tumor formation and regression for *Paqr8-OE* and control cells occurred with equivalent kinetics (Additional file 2: Fig. S2a). However, monitoring mice maintained off doxycycline revealed that *Paqr8-OE* tumors recurred significantly faster than controls (HR = 2.4, *p* = 0.003) (Fig. 2B). This indicates that Paqr8 is sufficient to accelerate the rate of tumor recurrence following Her2 downregulation.

Next, we conducted analogous experiments to determine the effects of *Paqr8* deletion (*Paqr8-KO*) on the rate of tumor recurrence. Two independent guide RNAs against *Paqr8* were validated using TIDE PCR [49], which demonstrated that > 90% of cells contained indels likely to result in loss-of-function mutations (Additional file 1: Fig. S1b, c). Cells transduced with either a guide RNA against *Paqr8* or *sgRosa* control were orthotopically injected into *nu/nu* mice maintained on doxycycline. Primary tumors derived from either *Paqr8-KO* or *sgRosa* control cells developed with equivalent kinetics and regressed to a non-palpable state following doxycycline removal and Her2 downregulation (Additional file 2: Fig. S2b). Kaplan–Meier analysis revealed that the rate of recurrence of *Paqr8-KO* tumors was markedly delayed compared to controls for each of the guides tested (HR = 0.26, *p* < 0.0001; HR = 0.29, *p* < 0.0001) (Fig. 2C). Together, these observations indicate that Paqr8 is both necessary and sufficient for efficient mammary tumor recurrence following downregulation of the Her2 pathway.

We next performed colony formation assays using the above *Paqr8-OE* and *Paqr8-KO* cells as an in vitro method to evaluate tumor cell outgrowth in the setting of Her2 inhibition. Colony formation in the presence of Her2 expression models primary tumor formation in mice, whereas colony formation in the absence of doxycycline and Her2 expression models Her2-independent tumor recurrence.

Consistent with our in vivo findings, we found no difference in the colony-forming ability of *Paqr8-OE* or *Paqr8-KO* cells and their respective controls in the presence of Her2 expression (Fig. 2D, E). In contrast, in the absence of Her2 expression *Paqr8-OE* cells formed a significantly greater number of colonies than controls (*p* = 0.003) (Fig. 2F). This parallels the increased rate of recurrence observed for *Paqr8-OE* tumor cells in mice. Conversely, in the absence of Her2 expression *Paqr8-KO* cells formed significantly fewer colonies than controls (*p* = 0.010) (Fig. 2G), paralleling the decreased rate of recurrence observed for *Paqr8-KO* tumor cells in mice.

To extend these findings to pharmacological inhibition of Her2 in human breast cancer cells, we overexpressed *PAQR8* in BT474-M1 cells, a Her2+/ER+ human breast cancer cell line. Consistent with our observations following the genetic downregulation of Her2, *PAQR8-OE* BT474-M1 cells treated with lapatinib, a small molecule Her2 tyrosine kinase inhibitor, exhibited increased viability compared to controls (Fig. 2H).

Together, the congruent effects of *Paqr8* overexpression and deletion in vivo and in vitro reinforce the conclusion that Paqr8 is both necessary and sufficient for tumor recurrence in the setting of Her2 inhibition, either by genetic downregulation or by pharmacological inhibition. In addition, these in vitro findings indicate that the effects of Paqr8 on Her2-independent growth are tumor cell autonomous, do not require stromal or host immune cells, and occur in both human and mouse cells.

### Paqr8 confers a competitive advantage on tumor cells following Her2 downregulation

**Fig. 3:**
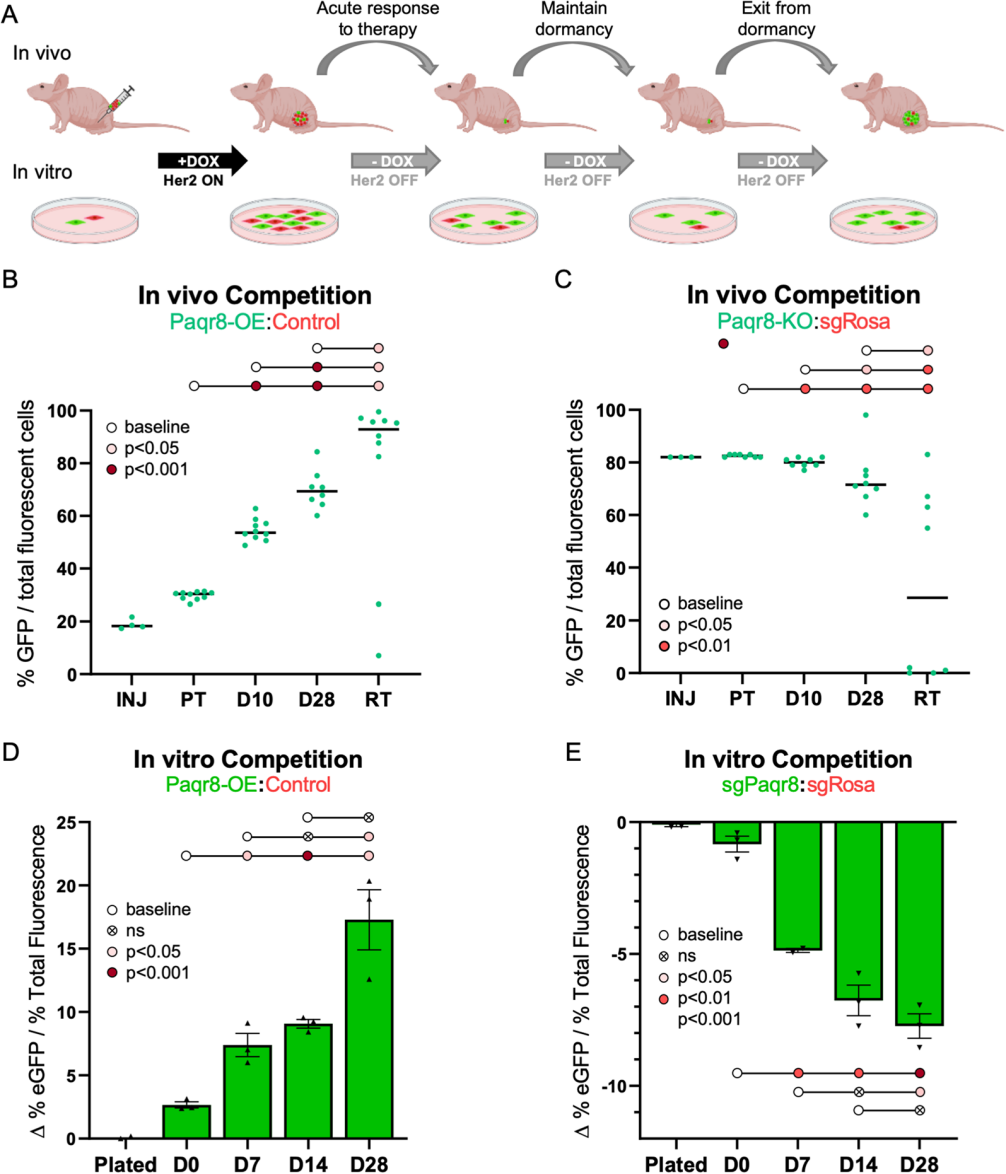
Paqr8 confers a competitive advantage to tumor cells following Her2 downregulation. **A** Experimental schema for in vivo and in vitro competition assays. **B** eGFP to mCherry ratios determined by ddPCR after injecting a mixture of 80% H2B-eGFP-labeled *Paqr8-OE* cells and 20% H2B-mCherry-labeled control cells. **C** eGFP to mCherry ratios determined by ddPCR after injecting a mixture of 20% eGFP-labeled *Paqr8-KO* and 80% mCherry-labeled *sgRosa* control cells. **D** eGFP to mCherry ratios determined by ddPCR after plating 50% H2B-eGFP-labeled *Paqr8-OE* cells and 50% H2B-mCherry-labeled control cells, following Her2 downregulation at time D0. Data were normalized to a control arm for which 50% H2B-eGFP-labled control cells and 50% H2B-mCherry-labeled control cells were plated in parallel. **E** eGFP to mCherry ratios determined by ddPCR from plating 50% eGFP-labeled *sgPaqr8* cells and 50% mCherry-labeled *sgRosa* control cells, following Her2 downregulation at time D0. Data were normalized to a control arm for which 50% eGFP-labeled *sgRosa* cells and 50% mCherry-labeled *sgRosa* cells were plated in parallel

Having demonstrated that Paqr8 is both necessary and sufficient for efficient breast cancer recurrence, we wished to identify the stages of tumor regression, cellular dormancy, and recurrence during which Paqr8 plays a role. To do so, we generated primary tumors by orthotopically injecting a mixture of 20% H2B-eGFP-labeled Her2-GEM model-derived *Paqr8-OE* cells and 80% H2B-mCherry-labeled vector control cells into *nu/nu* mice on doxycycline and then withdrew doxycycline to downregulate Her2 and induce tumor regression. We collected primary tumors (PT), dormant residual lesions at 10 or 28 days (D10, D28) following doxycycline withdrawal, and recurrent tumors (RT) arising in the setting of continued Her2 downregulation. These stages roughly correspond to acute tumor regression following Her2 downregulation (PT to D10), early-to-late tumor dormancy (D10 to D28), and tumor cell exit from dormancy and proliferation to yield recurrent tumors (D28 to RT) (Fig. 3A). At each stage, droplet digital PCR (ddPCR) using probes for eGFP and mCherry was used to quantify changes in the ratio of eGFP to mCherry-labeled cells.

Quantification of the eGFP to mCherry ratio following Her2 downregulation revealed progressive enrichment for eGFP-expressing *Paqr8-OE* cells across multiple stages of tumor progression, eventually resulting in recurrent tumors with > 90% *Paqr8-OE* cells (Fig. 3B). The greatest rate of enrichment for *Paqr8-OE* cells was observed from primary tumors to day 10 of tumor regression (*p* < 0.0001) (Fig. 3B), during which the bulk of tumor cells die following acute Her2 downregulation. Further selection for *Paqr8-OE* cells was observed during the dormant phase (D10 to D28) (*p* < 0.0001) and in recurrent tumors (*p* = 0.043) (Fig. 3B).

We next performed the converse experiment in which we orthotopically injected a mixture of 80% eGFP-labeled Her2 GEM model-derived *Paqr8-KO* cells and 20% mCherry-labeled *sgRosa* cells into *nu/nu* mice and collected primary tumors, residual lesions at D10 and D28 of dormancy, and recurrent tumors. Quantification of the ratio of eGFP to mCherry-labeled cells by ddPCR revealed that *Paqr8-KO* cells were progressively selected against, with significant depletion of *Paqr8-KO* cells observed acutely following therapy (PT to D10) (*p* = 0.002) (Fig. 3C). Selection against *Paqr8-KO* cells continued throughout dormancy (D10 to D28) (*p* = 0.010) and in recurrent tumors (*p* = 0.016) (Fig. 3C). Together, these results indicate that Paqr8 provides a selective advantage to tumor cells in vivo following acute Her2 downregulation, as well as during dormancy and tumor recurrence.

To further test this hypothesis, we performed in vitro competition assays (Fig. 3A). After plating a 50:50 mixture of eGFP-labeled *Paqr8-OE* or *Paqr8-KO* cells and their respective mCherry-labeled controls in the presence of doxycycline, cells were allowed to grow for three days. Cells were then collected prior to Her2 downregulation at Day 0 (D0) to model in vivo primary tumor growth, and at 7, 14, and 28 days (D7, D14, and D28) following doxycycline withdrawal during the period of dormancy. The ratio of eGFP to mCherry-labeled tumor cells was analyzed by ddPCR.

Following Her2 downregulation in vitro, *Paqr8-OE* cells were selected for, whereas *Paqr8-KO* cells were selected against, compared to controls (Fig. 3D, E). Significant positive selection for *Paqr8-OE* (*p* = 0.029), and negative selection against *Paqr8-KO* (*p* = 0.004), tumor cells occurred from D0 to D7, during which time cells undergo apoptosis in response to acute Her2 inhibition. Further selection for *Paqr8-OE*, and against *Paqr8-KO*, tumor cells was observed from D7 to D28 of the dormancy period. Thus, in vitro competition assays recapitulate results from in vivo studies in mice.

Together, these in vivo and in vitro findings consistently and concordantly indicate that Paqr8 confers a selective advantage on tumor cells following Her2 downregulation, particularly during the initial phase of response to therapy.

### Paqr8 promotes cell survival by reducing apoptosis following Her2 downregulation

**Fig. 4:**
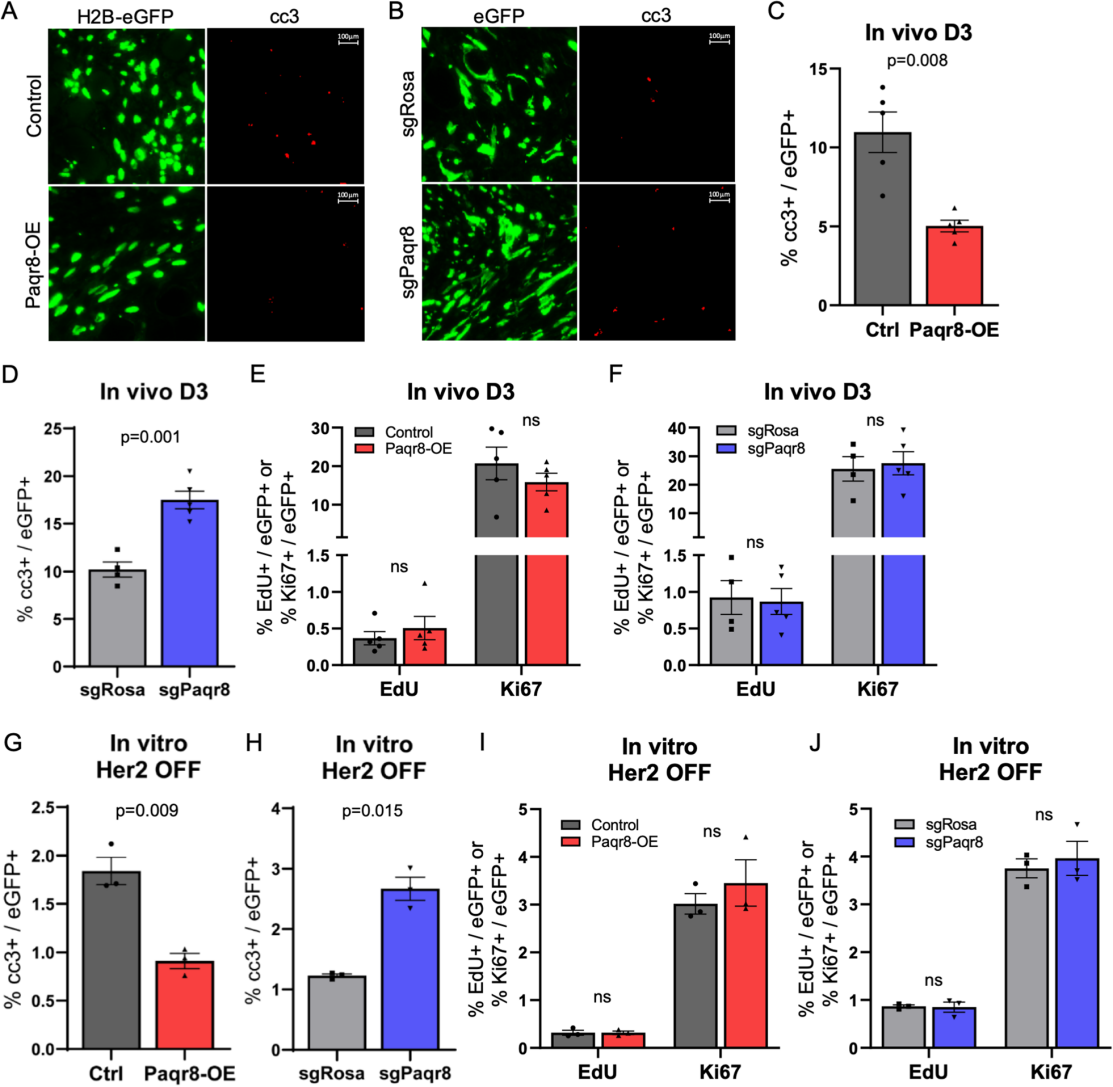
Paqr8 enhances cell survival without affecting proliferation following Her2 downregulation. Regressing tumors from *nu/nu* mice bearing *Paqr8-OE* or *Paqr8-KO* primary orthotopic tumors at three days (D3) following Her2 downregulation were stained by immunofluorescence for cleaved caspase-3 (cc3). Representative images are shown (**A**, **B**), and the proportions of eGFP + cells that were cc3 + were quantified (**C**, **D**). D3 regressing tumors were also stained for EdU and Ki67, and the proportions of eGFP + cells that were EdU + or Ki67 + were quantified (**E**, **F**). *Paqr8-OE* (**G**, **I**) or *Paqr8-KO* (**H**, **J**) cells were cultured in medium containing 1% serum without doxycycline (Her2 OFF) for 72 h. Cells were incubated with 10 mM EdU for 2 h prior to fixation, permeabilization, and staining by immunofluorescence. **G**, **H** Quantification of the proportion of eGFP + cells that were cc3+. **I**, **J** Quantification of the proportion of eGFP + cells that were EdU + or Ki67+

The competitive advantage exhibited by *Paqr8-OE*, and competitive disadvantage exhibited by *Paqr8-KO*, tumor cells following Her2 downregulation could potentially result from Paqr8-mediated differences in cell survival, proliferation, or both. We therefore performed immunofluorescence for markers for apoptosis (cleaved caspase-3), cell cycle (Ki67), and S-phase (EdU) in *Paqr8-OE* and *Paqr8-KO* tumor cells following Her2 downregulation in vivo and in vitro. In vivo, *Paqr8-OE* cells exhibited significantly reduced levels of staining for cleaved caspase-3 compared to controls 72 h after Her2 downregulation (*p* = 0.008) (Fig. 4A, C), whereas *Paqr8-KO* cells exhibited significantly increased levels of cleaved caspase-3 staining compared to controls (*p* = 0.001) (Fig. 4B, D). Similarly, 72 h after Her2 downregulation in vitro, *Paqr8-OE* cells exhibited significantly lower levels of cleaved caspase-3 compared to controls (*p* = 0.009) and *Paqr8-KO* cells exhibited significantly higher levels of cleaved caspase-3 compared to controls (*p* = 0.015) (Fig. 4G, H).

No significant differences were observed in staining for EdU or Ki67 between *Paqr8-OE* and *Paqr8-KO* cells and their respective controls either in vivo (Fig. 4E, F; Additional file 3: Fig. S3) or in vitro (Fig. 4I, J; Additional file 4: Fig. S4). In addition, in the presence of Her2 expression, both *Paqr8-OE* and *Paqr8-KO* tumor cells exhibited levels of staining for apoptotic and proliferative markers that were similar to their respective controls (Additional file 5: Fig. S5).

In aggregate, these data indicate that Paqr8 promotes cell survival following Her2 downregulation without exerting a significant impact on cell proliferation. This, in turn, suggests that the competitive advantage conferred on tumor cells by Paqr8 following Her2 downregulation is principally mediated by effects of Paqr8 on tumor cell survival.

### PAQR8 promotes survival of ER+ tumor cells following estrogen pathway inhibition in vitro

**Fig. 5:**
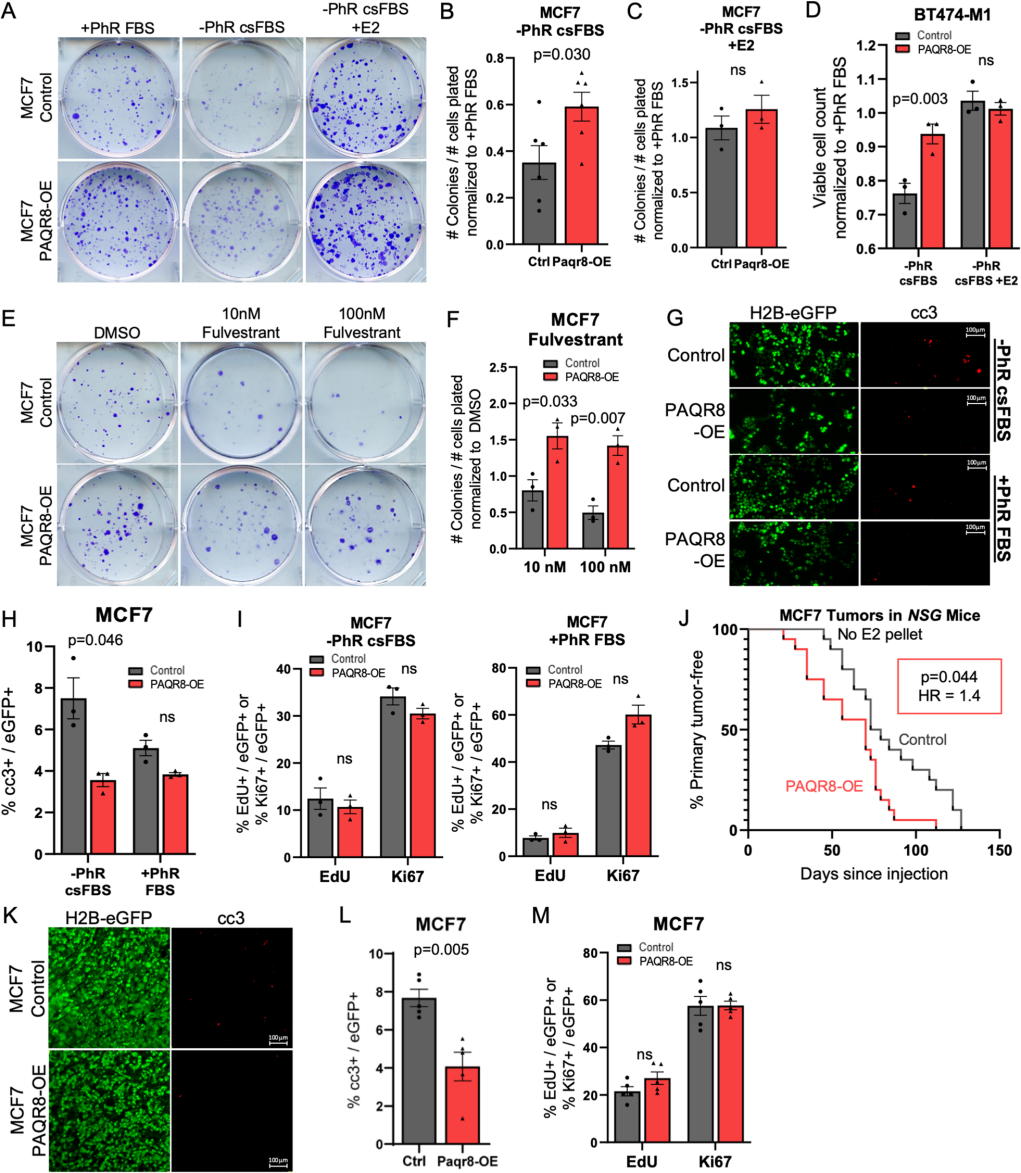
Paqr8 promotes ER+ breast cancer cell survival following E2 pathway inhibition. **A** Representative images of MCF7 colony formation assays performed in growth medium (+ PhR FBS), E2-deprived medium (–PhR csFBS), or E2-deprived medium supplemented with 1 nM E2 (–PhR csFBS + E2). Proportion of cells plated that formed colonies in –PhR csFBS (**B**) or –PhR csFBS + E2 medium (**C**), normalized to + PhR FBS medium. **D** Viable cell count of BT474-M1 cells in **–**PhR csFBS or –PhR csFBS + E2 medium, normalized to + PhR FBS medium. **E** Representative images of MCF7 colony formation assays performed in the presence of increasing concentrations of fulvestrant or vehicle control (DMSO), and quantification (**F**). **G** Representative images of immunofluorescence staining for cleaved caspase-3 (cc3) in –PhR csFBS or + PhR FBS medium, and quantification (**H**). **I** Percentage of eGFP+ cells that were EdU+ or Ki67+ following 2 h of 10 mM EdU labeling. (**J**) Time to first palpation of MCF7 tumors forming in *NSG* mice without E2 pellet supplementation. **K** Representative cc3 staining in primary tumor tissue sections, and quantification (**L**). Sections were also stained for EdU and Ki67, and the percentages of eGFP+ cells that were EdU+ or Ki67+ were quantified (**M**)

The finding that *PAQR8* CN gain was mutually exclusive with activating *ESR1* mutations among patients treated with anti-estrogen therapy (Fig. 1A) raised the intriguing hypothesis that PAQR8 might play a role in promoting resistance to anti-estrogen therapy in a manner that is in some way redundant with *ESR1* mutation [3]. To test this hypothesis, we overexpressed *PAQR8* in MCF7 cells, an ER+ human breast cancer cell line with low endogenous expression of PAQR8. Under complete growth conditions, comprised of 10% fetal bovine serum (FBS) in base medium containing the estrogenic pH indicator phenol red (PhR) [50], *PAQR8-OE* MCF7 cells exhibited a modestly increased ability to form colonies (Fig. 5A). However, under conditions of estrogen deprivation achieved using charcoal-stripped serum (csFBS) and PhR-free medium, the magnitude of this PAQR8-dependent enhancement of colony-forming capacity was increased (*p* = 0.030) (Fig. 5A, B; Additional file 6: Fig. S6a).

Since charcoal-stripped serum lacks not only estrogen but also other lipid-soluble constituents, we reintroduced 17β-estradiol (E2) to csFBS PhR-free medium to determine whether the observed effects were solely due to estrogen removal. Indeed, addition of 1 nM E2 to csFBS PhR-free medium abrogated the increased colony formation conferred by PAQR8 overexpression under estrogen-deprived conditions (Fig. 5A, C; Additional file 6: Fig. S6a).

Consistent with our findings in MCF7 cells, PAQR8 overexpression in Her2+/ER+ BT474-M1 cells also conferred resistance to estrogen deprivation in csFBS PhR-free medium (*p* = 0.003) that was rescued by the addition of estradiol (Fig. 5D; Additional file 6: Fig. S6b).

Furthermore, we found that MCF7 *PAQR8-OE* cells exhibited increased colony formation in the presence of fulvestrant, a selective estrogen receptor degrader (Fig. 5E, F; Additional file 6: Fig. S6c). Overall, these findings indicate that PAQR8 confers resistance to multiple forms of estrogen receptor pathway inhibition.

To evaluate whether the enhanced colony-forming capacity of *PAQR8-OE* MCF7 cells under E2-deprived media conditions was due to cell survival, cell proliferation, or both, we performed immunofluorescence for cleaved caspase-3, EdU, and Ki67 in cells cultured under estrogen-deprived or estrogen-replete conditions. *PAQR8-OE* cells displayed significantly lower levels of cleaved caspase-3 staining compared to controls under E2-deprived conditions (*p* = 0.046), but not under E2-replete conditions (Fig. 5G, H). In contrast, the percentage of cells that were EdU+ or Ki67+ did not differ significantly between MCF7 *PAQR8-OE* cells and controls under either condition (Fig. 5I; Additional file 7: Fig. S7).

Consistent with the conclusion from the above findings that PAQR8 promotes cell survival in the setting of therapy targeting either Her2 or ER, rather than mediating an effect of estrogen per se, we found that addition of either estradiol or progesterone to ER-negative Her2-dependent primary mouse cells in charcoal-stripped serum had no effect on their viability, irrespective of *PAQR8* overexpression or deletion (Additional file 8: Fig. S8).

### *PAQR8 promotes survival of ER*+ *tumor cells following estrogen deprivation *in vivo

To determine whether PAQR8-dependent effects on cell survival observed under E2-deprived media conditions in vitro were recapitulated in vivo, we orthotopically injected MCF7 *PAQR8-OE* or control cells into NOD SCID gamma (*NSG*) mice. MCF7 cells have been shown to form tumors in *NSG* mice without the need for E2 pellet supplementation [51]. *NSG* mice also exhibit low endogenous levels of E2 compared to human beings [52], hence modeling conditions of estrogen deprivation.

Following orthotopic injection, mice were monitored for primary tumor formation. MCF7 cells overexpressing *PAQR8* formed detectable tumors significantly earlier than vector controls (HR = 1.4, *p* = 0.044) (Fig. 5J). When tumors reached 8 × 8 mm, mice were sacrificed, and tumors were fixed, sectioned, and stained by immunofluorescence for apoptotic and proliferative markers. We found that *PAQR8-OE* tumors displayed significantly reduced levels of cleaved caspase-3 compared to control tumors of similar size (*p* = 0.005) (Fig. 5K, L). In contrast, no significant difference was observed between *PAQR8-OE* tumors and controls with respect to the percentage of tumor cells that were EdU+ or Ki67+ (Fig. 5M; Additional file 9: Fig. S9). Thus, consistent with in vitro findings under estrogen-deprived conditions, PAQR8 also confers a survival advantage in response to estrogen deprivation in vivo.

### PAQR8 confers resistance to chemotherapies

As noted above, *PAQR8* CN gain in recurrent breast cancers was not restricted to hormone receptor-positive tumors and occurred with equal frequency in recurrent metastatic tumors in patients treated with other forms of therapy, including chemotherapy and anti-Her2 therapy [3]. This suggested the possibility that PAQR8 might promote resistance to therapies beyond those targeting ER or Her2.

**Fig. 6:**
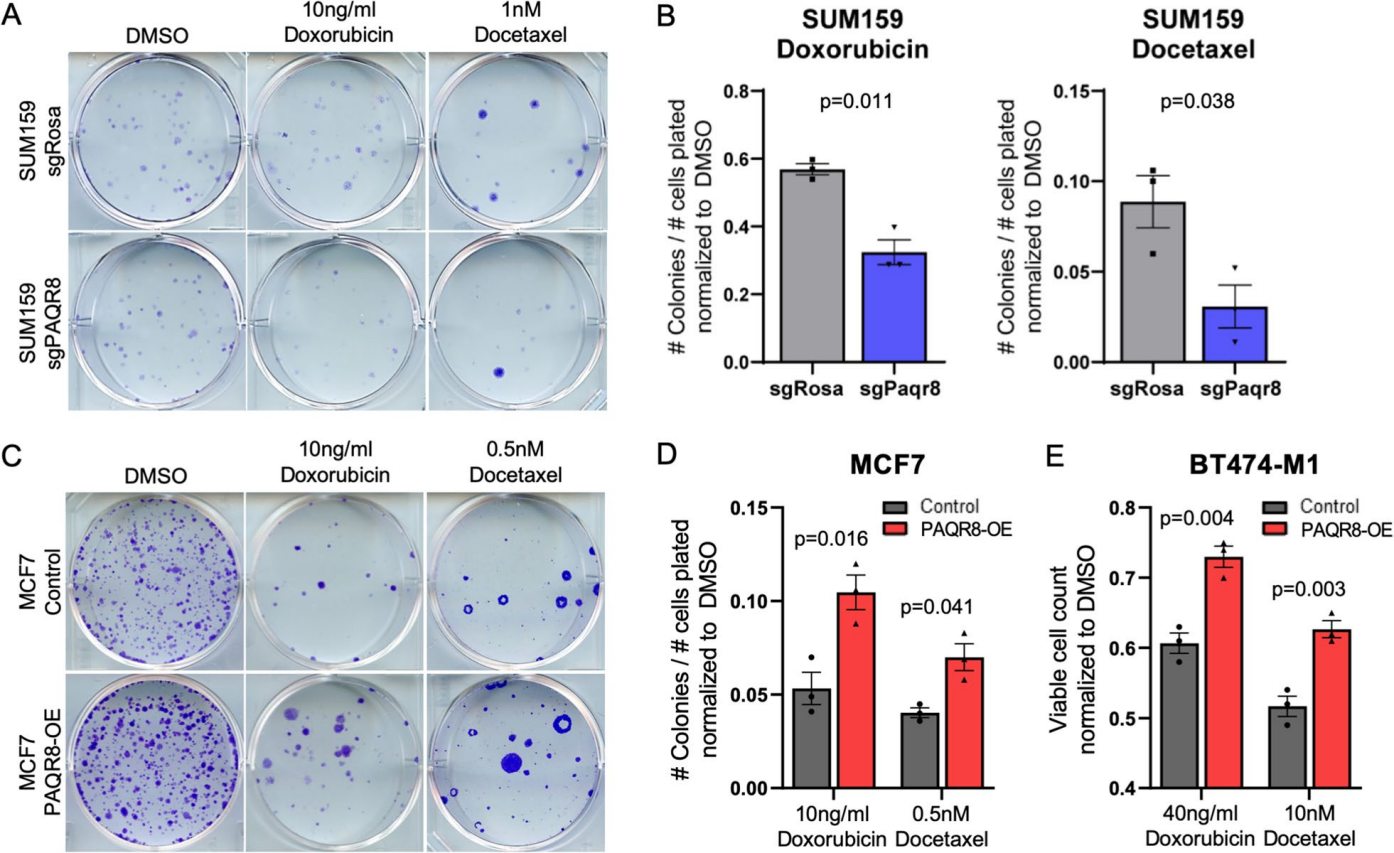
PAQR8 promotes resistance to chemotherapy. **A** Representative images of colony formation assays of SUM159 cells in media containing doxorubicin, docetaxel, or vehicle control (DMSO). **B** Proportion of plated cells that formed colonies in the presence of doxorubicin or docetaxel, normalized to DMSO control. **C** Representative images of colony formation assays of MCF7 cells in media containing doxorubicin, docetaxel, or vehicle control (DMSO). **D** Proportion of plated cells that formed colonies in the presence of doxorubicin or docetaxel, normalized to DMSO control. **E** Viable cell counts of BT474-M1 cells in the presence of doxorubicin or docetaxel, normalized to DMSO control

To investigate this hypothesis, we tested the effects of PAQR8 overexpression or *PAQR8* deletion on the response to treatment with doxorubicin or docetaxel, chemotherapeutic agents commonly used in treating breast cancer patients. We first used CRISPR-cas9 to delete *PAQR8* in the triple-negative human breast cancer cell line SUM159, which expresses high endogenous levels of *PAQR8*. Colony formation assays were then performed in which *PAQR8-KO* and control SUM159 cells were treated with doxorubicin or docetaxel. This revealed that *PAQR8-KO* SUM159 cells exhibited increased sensitivity to both doxorubicin and docetaxel compared to controls (Fig. 6A, B; Additional file 10: Fig. S10a, b).

Consistent with this result, MCF7 cells overexpressing PAQR8 exhibited increased colony formation compared to controls in the presence of either doxorubicin or docetaxel (Fig. 6C, D; Additional file 10: Fig. S10c). Similarly, BT474-M1 cells overexpressing PAQR8 exhibited increased viability compared to controls in the presence of either doxorubicin or docetaxel (Fig. 6e; Additional file 10: Fig. S10d). Together, these findings suggest that PAQR8 promotes breast cancer cell resistance to chemotherapy.

### Pro-survival effects of PAQR8 are mediated by a G_i_-dependent reduction in cAMP

Endogenous PAQR8 and its related mPR family member PAQR7 have been reported to co-immunoprecipitate with G_i_ proteins in human myometrial cells [11]. Consistent with G_i_ protein-mediated effects, *Paqr7* siRNA knockdown abrogated the progesterone-induced reduction in cAMP levels in mouse neuronal cells [18]. In contrast, siRNA knockdown of Paqr8 in mouse neuronal cells did not affect progesterone-induced changes in cAMP levels [18]. Hence, the question of whether PAQR8 functions by coupling to G_i_ proteins remains unclear.

**Fig. 7:**
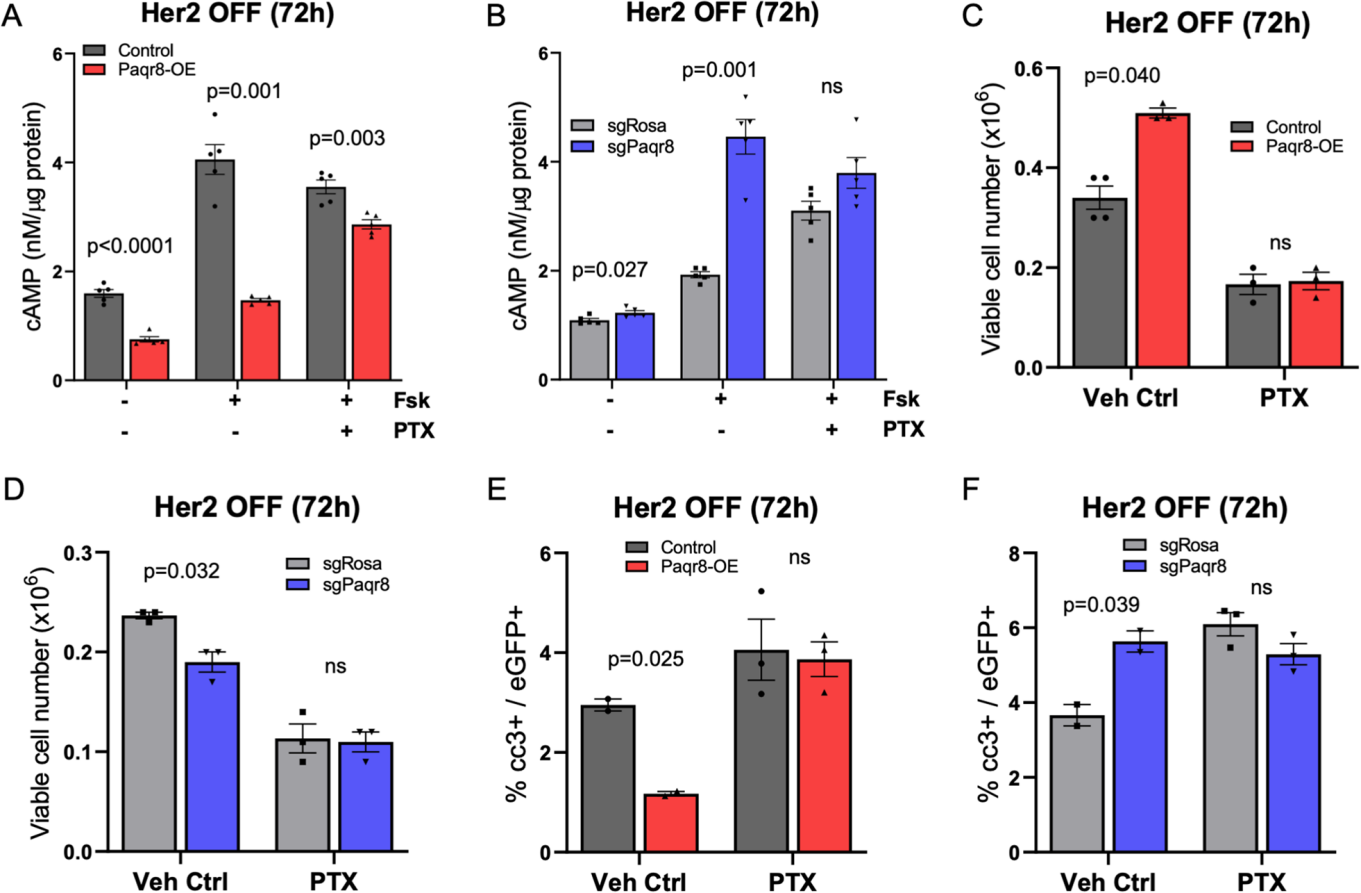
Cell survival effects of Paqr8 are dependent on Gi protein-mediated reductions in cAMP. **A**, **B** Intracellular cAMP measurements of Her2-dependent primary mouse tumor cells following 72 h of Her2 downregulation, with or without addition of 1μM forskolin (Fsk) for 15 min prior to collection, and with or without 12-h preincubation with 100 ng/ml pertussis toxin (PTX). **C**, **D** Viable cell counts of Her2-dependent primary tumor cells at 72 h following Her2 withdrawal, in the presence or absence of 100 ng/ml PTX. **E**, **F** Percentage of eGFP+ cells that were cleaved caspase-3 + by immunofluorescence staining of Her2-dependent primary cells cultured for 72 h after Her2 downregulation, in the presence or absence of 100 ng/ml PTX

To address this, we measured cAMP levels in mouse *Paqr8-OE* and *Paqr8-KO* Her2-dependent primary tumor cells following acute Her2 downregulation, conditions under which Paqr8 exerts a pro-survival effect. Following Her2 downregulation for 72 h, Paqr8 overexpression reduced basal, as well as forskolin-stimulated, levels of cAMP compared to control cells (*p* = 2.72e-05, *p* = 0.001) (Fig. 7A). Conversely, under the same conditions, *Paqr8-KO* cells exhibited increased basal, as well as forskolin-stimulated, levels of cAMP compared to controls (*p* = 0.027, *p* = 0.001) (Fig. 7B). These data suggest that Paqr8 reduces cAMP levels in the setting of Her2 inhibition.

To determine whether the observed changes in cAMP levels were mediated by a G_i_ protein-coupled receptor, we preincubated *Paqr8-OE* or *Paqr8-KO* tumor cells with pertussis toxin (PTX) prior to measuring forskolin-stimulated levels of cAMP. PTX preincubation for 12 h abrogated Paqr8-dependent increases in cAMP in *Paqr8-KO* cells stimulated with forskolin and markedly reduced Paqr8-dependent decreases in cAMP in *Paqr8-OE* cells, compared to controls (Fig. 7A, B). This indicates that Paqr8-mediated changes in cAMP levels are dependent on a G_i_ protein-coupled receptor.

Next, we investigated whether the pro-survival effects of Paqr8 are also dependent on its G_i_ protein-coupled activity. Consistent with our prior results, we observed an increase in viable *Paqr8-OE* tumor cells, as well as a decrease in viable *Paqr8-KO* tumor cells, compared to controls following Her2 downregulation for 72 h (Fig. 7C, D). Notably, co-incubation with PTX abrogated differences in viable cell number between *Paqr8-OE* and *Paqr8-KO* cells and their respective controls following Her2 downregulation (Fig. 7C, D).

To confirm and extend this finding, we performed immunofluorescence for cleaved caspase-3 following Her2 downregulation in the presence of PTX. As before, Paqr8 overexpression resulted in a reduced percentage of cleaved caspase-3-positive cells (*p* = 0.025), whereas *Paqr8* deletion resulted in an increased percentage of cleaved caspase-3-positive cells (*p* = 0.039), 72 h after Her2 downregulation (Fig. 7E, F). In contrast, no differences in cleaved caspase-3 staining were observed between *Paqr8-OE* cells and controls, or between *Paqr8-KO* cells and controls, in the presence of PTX (Fig. 7E, F). Together, these studies indicate that the pro-survival effects conferred by Paqr8 in the setting of acute Her2 inhibition are dependent on a reduction in cAMP levels mediated by a G_i_ protein-coupled receptor.

### Paqr8 alters the balance of ceramides and sphingosine-1-phosphate

Beyond the hypothesis that PAQR8 functions as a GPCR, it has been suggested that PAQR8 might function as an alkaline ceramidase, or possibly as both a ceramidase and a GPCR [22]. Alkaline ceramidases convert ceramides to sphingosine, which in turn can be phosphorylated by sphingosine kinase to produce sphingosine-1-phosphate (S1P) [53]. While ceramides promote apoptosis, S1P can suppress apoptosis, in part by counterbalancing pro-apoptotic effects of ceramides [54]. Thus, the interconversion between ceramides and S1P, referred to as the ceramide:S1P ‘rheostat’, plays an important role in determining the cellular balance between survival and death [55].

**Fig. 8:**
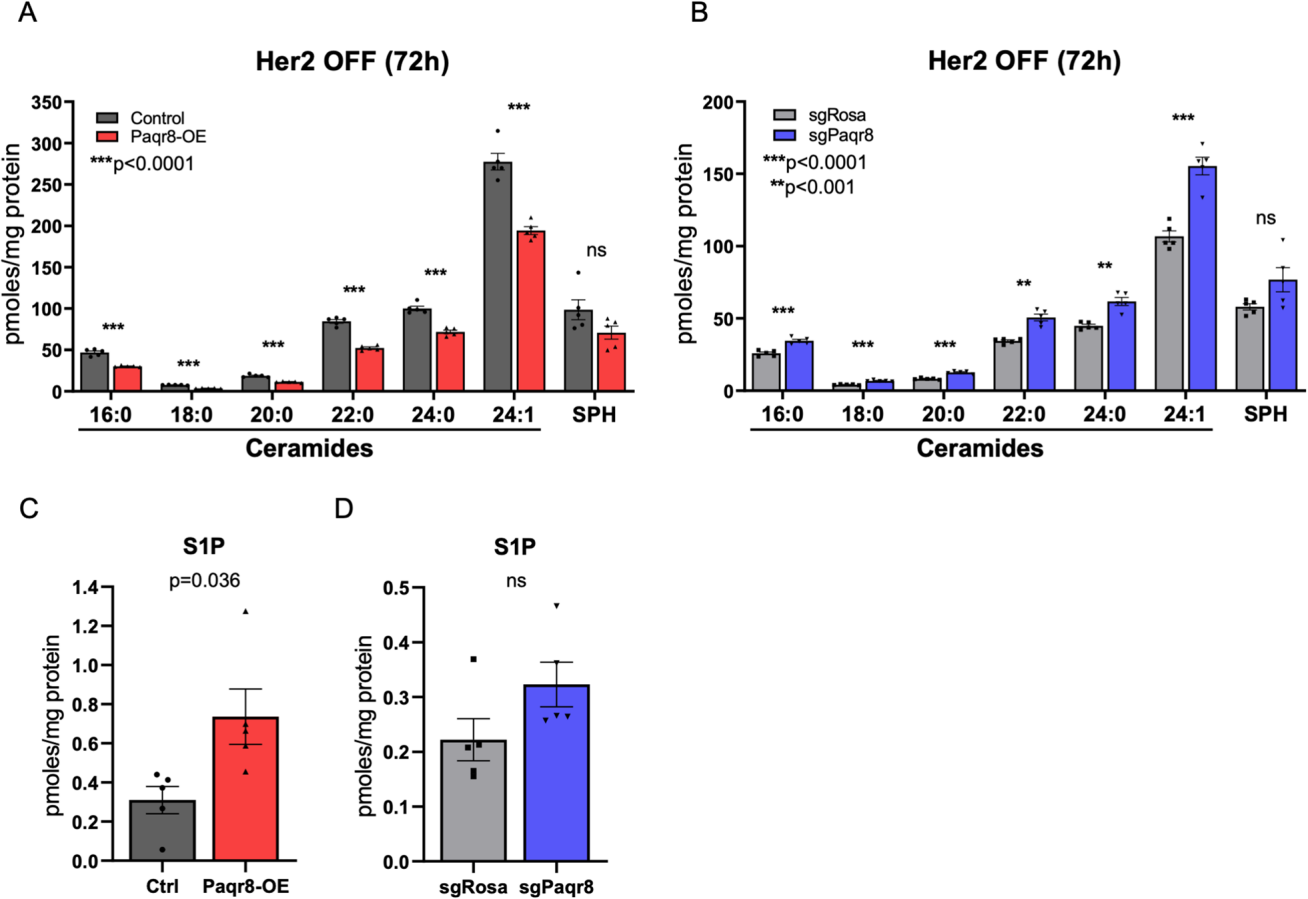
Paqr8 decreases ceramide levels and increases S1P levels. **A**, **B** Levels of ceramides Cer(d18:1_16:0, 18:0, 20:0, 22:0, 24:0, 24:1) and sphingosine (SPH) in Her2-dependent primary mouse tumor cells quantified by LC-HRMS 72 h after Her2 downregulation. **C**, **D** Sphingosine-1-phosphate (S1P) levels quantified in the same cells by LC-HRMS 72 h after Her2 downregulation

To begin to query whether PAQR8 might function as a ceramidase, we analyzed the sphingolipidome of *Paqr8-OE* and *Paqr8-KO* Her2-dependent primary tumor cells 72 h after Her2 downregulation using liquid chromatography-high resolution mass spectrometry (LC-HRMS). *Paqr8-OE* cells exhibited significantly lower levels of the most abundant ceramides Cer(16:0, 18:0, 20:0, 22:0, 24:0, and 24:1) (Fig. 8A). Conversely, *Paqr8-KO* cells exhibited significantly higher levels of these same ceramide species compared to controls (Fig. 8B). These data are consistent with increased ceramidase activity in Paqr8 expressing cells, which would hydrolyze and thereby reduce ceramide levels within cells.

Interestingly, significant changes in levels of sphingosine, the product of ceramide hydrolysis, were not observed in either *Paqr8-OE* or *Paqr8-KO* cells compared to controls (Fig. 8A, B). Sphingosine can be phosphorylated by sphingosine kinases to form sphingosine-1-phosphate (S1P), which counterbalances the pro-apoptotic effects of ceramides and sphingosine. Consistent with this mode of regulation, cellular S1P levels were significantly increased in *Paqr8-OE* cells compared to controls (*p* = 0.036) (Fig. 8C). A significant difference in S1P levels between *Paqr8-KO* cells compared to *sgRosa* controls was not observed (Fig. 8D). As S1P levels are generally 100–1000-fold lower than ceramide levels, this finding may be attributable to limitations in assay sensitivity.

Together, our findings indicate that Paqr8 expression decreases ceramide levels, increases S1P levels, and thereby decreases the ceramide:S1P ratio, which would be anticipated to favor cell survival. Further, the observed Paqr8-dependent decreases in ceramides are consistent with a model in which Paqr8 can function as an alkaline ceramidase, and further suggest that a Paqr8-mediated reduction in the ratio of ceramide:S1P may underlie its pro-survival effects.

## Discussion

Although therapy-resistant recurrent disease is principally responsible for breast cancer mortality, mechanisms of resistance have been identified in only a minority of cases. This is exemplified by resistance to anti-estrogen therapies, which ultimately develops in most, if not all, metastatic breast cancers treated with such agents. While *ESR1* activating mutations, MAPK pathway alterations, and mutations in *MYC* and other transcription factors are known mechanisms of resistance in hormone receptor-positive breast cancer, it has been estimated that these mechanisms together account for only 40% of endocrine resistance [56]. Elucidating the, as yet, ‘occult’ mechanisms that may account for ~ 60% of endocrine therapy resistance is therefore a critical priority.

We identified *PAQR8* CN gain as a common focal CN alteration that preferentially occurs in therapy-resistant recurrent metastatic tumors in breast cancer patients [3]. Our findings that *PAQR8* CN gain was mutually exclusive with activating *ESR1* mutations in patients treated with anti-estrogen therapy, and that *PAQR8* CN gain occurred with equal frequencies in patients who received anti-estrogen therapy and in those treated with chemotherapy or agents targeting Her2 [3], suggested the possibility that PAQR8 might confer resistance to multiple forms of therapy. This hypothesis is further supported by our findings here that *Paqr8* is spontaneously upregulated and frequently undergoes CN gain in recurrent mammary tumors in mice arising from Her2, Akt, Myc, and Wnt1-induced primary tumors subjected to oncogene downregulation, which is a genetic surrogate for targeted therapy.

Consistent with our findings of frequent *PAQR8* CN gain in therapy-resistant recurrent tumors in breast cancer patients and mouse models, we now provide the first in vivo evidence that PAQR8 plays a functional role in cancer and does so through its ability to confer a survival advantage on tumor cells subjected to different forms of therapy. In particular, we demonstrated that PAQR8 is both necessary and sufficient to promote efficient mammary tumor recurrence following Her2 inhibition and that PAQR8 facilitates the outgrowth of orthotopically implanted ER + tumor cells under conditions of estrogen deprivation. These in vivo tumor-promoting effects of PAQR8 were attributable to its ability to promote tumor cell survival in response to Her2 downregulation or pharmacologic inhibition, estrogen deprivation or anti-estrogen therapy, or treatment with the chemotherapeutic agents doxorubicin or docetaxel. Together, these observations reveal a role for PAQR8 in mediating resistance to multiple types of therapy, which in turn can promote tumor recurrence.

While *ESR1* mutations are the most common mechanism of endocrine resistance identified to date, they occur in only ~20% of treatment-resistant tumors and are generally limited to patients treated with aromatase inhibitors [7, 8, 57, 58]. Thus, even after considering mutations in *ESR1* and other known mechanisms, nearly 60% of endocrine resistance is unexplained [56]. Accordingly, our observations that *PAQR8* CN gain occurs in > 50% of patients treated with anti-estrogen therapies, as well as in > 50% of patients treated with chemotherapy or Her2-targeted therapies [3], suggest the important possibility that PAQR8 upregulation may account for a substantial fraction of therapy resistance, including endocrine therapy resistance, and in doing so may facilitate tumor recurrence across multiple subtypes of breast cancer.

PAQR8 has been reported to function as a membrane progesterone receptor that conveys rapid non-genomic effects of progesterone signaling. Our findings presented here, however, indicate that PAQR8 can function in the absence of progesterone. Indeed, in ER/PR-negative Her2-dependent mouse primary tumor cells, the addition of progesterone to charcoal-stripped serum, which is depleted of lipid-soluble hormones such as progesterone, did not affect the ability of Paqr8 overexpression to enhance, or *Paqr8* deletion to impair, cell viability following Her2 downregulation. Moreover, both the increased colony-forming ability and survival advantage that we observed for PAQR8 overexpressing MCF7 cells were enhanced, not diminished, in charcoal-stripped serum.

Our findings that PAQR8 can function independently of progesterone are consistent with a study in yeast that employed a heterologous reporter construct containing a portion of the *FET3* promoter that is repressed by the yeast PAQR protein Izh2p*.* In this system, human PAQR7 required progesterone for repression of *FET3* reporter activity, whereas PAQR8 repressed this construct in the absence of progesterone, as did several other PAQR family members [19]. Thus, the extent to which progesterone-dependent and independent effects of PAQR8 may be context-dependent, or may differ from those of PAQR7, remains to be clarified.

Based on the presence of three motifs conserved among alkaline ceramidases, PAQR8 has been hypothesized to possess ceramidase activity [22]. Indeed, PAQR1 and PAQR2 (i.e., adiponectin receptors 1 and 2) have been reported to possess low levels of basal ceramidase activity [25]. However, evidence in the literature that mPRs in eukaryotic cells possess ceramidase activity is lacking [17]. We provide the first functional evidence to support the hypothesis that PAQR8 may function as a ceramidase by demonstrating that knockdown of endogenous PAQR8 increases ceramide levels, whereas PAQR8 overexpression decreases ceramide levels while increasing levels of S1P.

Ceramides are established mediators of apoptosis and can act via a diverse array of mechanisms, including inhibition of Bcl-2, Bcl-xL the PI3K/AKT pathway, and pathways involving hnRNPA1, a regulator of alternative splicing [59–61]. Conversely, S1P is an inhibitor of ceramide-mediated apoptosis [62]. Thus, PAQR8 function as a ceramidase would be consistent with the pro-survival effects that we observe. Nevertheless, these PAQR8-dependent changes in sphingolipidome composition could be directly mediated by PAQR8 or by activation of a downstream ceramidase. Hence, the mechanisms by which PAQR8 affects sphingolipid metabolism warrant further investigation.

At the molecular level, we determined that the pro-survival effects of PAQR8 were mediated by a G_i_ protein-dependent reduction in cAMP levels that was abrogated by treatment with pertussis toxin. G_i_ protein activation can mediate anti-apoptotic effects via activation of MAPK or PI3K/AKT pathways, resulting in the inhibition of caspases and pro-apoptotic proteins such as BAX and BAD [63–65]. Indeed, PAQR8 and G_i_ have been reported to co-immunoprecipitate [11] and to be located within 40 nm of each other as assessed by an in situ proximity ligation assay [17], as would be anticipated if PAQR8 functions as a GPCR.

An alternative model to explain our findings would be if PAQR8 functions as a ceramidase that facilitates conversion of ceramides to the downstream sphingosine metabolite, S1P. S1P binding to S1P receptors, which are GPCRs [66], could in turn activate G_i_ proteins, resulting in anti-apoptotic effects mediated by ERK1/2 activation or suppression of BAX expression [67–69]. An additional possibility is that PAQR8 may function as both a ceramidase and a GPCR [22]. In this regard, recent computational predictions suggest that PAQR8 may possess eight transmembrane domains [70, 71], unlike the 7-transmembrane structure of ceramidases and GPCRs, such that the additional C-terminal transmembrane domain could potentially couple to a G_i_ protein. However, no experimental evidence to date supports such a structure. Further investigation will be required to clarify the direct or indirect nature of PAQR8-dependent effects on ceramide levels as well as G_i_ protein activation.

## Conclusions

In aggregate, our studies provide in vivo evidence that PAQR8 plays a functional role in cancer, implicate PAQR8, ceramide metabolism, and cAMP in breast cancer recurrence, and identify a novel mechanism of resistance to multiple antineoplastic therapies. In addition, the possibility that PAQR8 may contribute to a substantial fraction of treatment resistance in breast cancer patients nominates this poorly understood molecule for further study as a potential therapeutic target to reverse therapy resistance and improve outcomes for breast cancer patients.

## Supplementary Information

**Additional file 1:** Figure S1. Validating PAQR8/Paqr8 overexpression and knockout. a Western blot showing anti-HA tagged PAQR8/Paqr8 and anti-β-tubulin loading control. b Synthego ICE analysis results for Paqr8 knockout cells showing guide target sequence, PAM sequence, indel %, and knockout score. c TIDE sequencing results showing relative proportion of specific indels.

**Additional file 2:** Paqr8 does not affect the rate of primary tumor growth in the presence of Her2 expression. *nu/nu* mice were orthotopically injected with Her2-dependent primary mouse cells. Doxycycline in the drinking water maintained Her2 expression during primary tumor formation. Time from injection to palpation of 5x5mm primary tumors is shown.

**Additional file 3:** Paqr8 does not affect cell proliferation following acute Her2 withdrawal in vivo. *nu/nu* mice harboring Paqr8-OE or Paqr8-KO tumors from orthotopically injected Her2-dependent primary mouse cells three days following Her2 downregulation. Mice were injected with 50mg/kg EdU (i.p.) 2h prior to sacrifice. Residual lesions were harvested, sectioned, and stained by immunofluorescence for EdU and Ki67. Representative images are shown.

**Additional file 4:** Paqr8 does not affect proliferation following acute Her2 downregulation in vitro. Her2-dependent primary mouse cells were cultured in 1% serum without doxycycline (Her2 OFF) for 72h and incubated with 10mM EdU for 2h prior to fixation, permeabilization, and staining for EdU and Ki67. Representative images are shown.

**Additional file 5:** Paqr8 does not affect rates of apoptosis or cell proliferation in the presence of Her2 expression in vivo or in vitro. *nu/nu* mice harboring 5x5mm primary tumors from orthotopically injected Her2-dependent Paqr8-OE (a, c) or Paqr8-KO (b, d) primary mouse tumor cells were injected with 50mg/kg EdU (i.p.) 2h prior to sacrifice. Primary tumors were harvested, sectioned, and stained by immunofluorescence for cleaved caspase-3 (cc3), EdU, and Ki67. a-d Percentage of eGFP+ cells that were cc3+, EdU+, or Ki67+ as indicated. Paqr8-OE (e, g) or Paqr8-KO (f, h) Her2-dependent primary cells were cultured in the presence of 10% serum with doxycycline (Her2 ON) and incubated with 10mM EdU for 2h prior to fixation, permeabilization, and staining for cc3, EdU, and Ki67. e-h Percentage of eGFP+ that were cc3+, EdU+, or Ki67+, as indicated.

**Additional file 6:** a Proportion of plated MCF7 cells that formed colonies in +PhR csFBS, -PhR csFBS, or -PhR csFBS +E2 medium. b Viable cell count of BT474-M1 cells in +PhR FBS, -PhR csFBS, or -PhR csFBS +E2 medium. c Proportion of MCF7 cells plated that formed colonies in fulvestrant or vehicle control (DMSO).

**Additional file 7:** PAQR8 does not affect proliferation of MCF7 cells following 72h of estrogen deprivation in vitro. MCF7 cells were cultured in either growth medium containing phenol red and fetal bovine serum (+PhR FBS) or estrogen-deprived medium without phenol red containing charcoal-stripped fetal bovine serum (-PhR csFBS) for 72h. Cells were incubated with 10mM EdU for 2h prior to fixation, permeabilization, and immunofluorescence staining for EdU and Ki67. Representative images are shown.

**Additional file 8:** Effects of Paqr8 on viable cell count following 72h of Her2 withdrawal do not depend on presence of progesterone or estrogen. Her2-dependent primary mouse tumor cells were cultured without doxycycline (Her2 OFF) for 72h. Media contained 1% charcoal-stripped FBS (csFBS), in the presence of 1mM progesterone (P4), 1nM estrogen (E2), or neither.

**Additional file 9:** PAQR8 does not affect proliferation of MCF7 cells in primary tumors formed in NSG mice without estrogen supplementation. NSG mice harboring orthotopic MCF7 tumors were injected with 50mg/kg of EdU (i.p.) 2h prior to sacrifice. Tumors were harvested and fixed in 4% paraformaldehyde, paraffin embedded, sectioned and stained by immunofluorescence for EdU and Ki67. Representative images are shown.

**Additional file 10:** a Proportion of plated SUM159 cells that formed colonies in the presence of doxorubicin or vehicle control (DMSO). b Proportion of plated SUM159 cells that formed colonies in the presence of docetaxel or vehicle control (DMSO). c Proportion of plated MCF7 cells that formed colonies in the presence of doxorubicin, docetaxel, or vehicle control (DMSO). d Viable cell counts of BT474-M1 cells in the presence of doxorubicin, docetaxel, or vehicle control (DMSO).

**Additional file 11:** Full western blot image corresponding to Figure S1a.

## Data Availability

The datasets used in the current study are available from the corresponding author on reasonable request.

## Abbreviations

CN: Copy number
cAMP: Cyclic adenosine monophosphate
ER: Estrogen receptor
mPR: Membrane progesterone receptor
PAQR: Progestin and adipoQ receptor
GPCR: G protein-coupled receptor
G_i_: Inhibitory G protein
PTX: Pertussis toxin
TCGA: The Cancer Genome Atlas
GEM: Genetically engineered mouse
OE: Overexpression
KO: Knock-out
*nu/nu*: Athymic nude
PT: Primary tumor
RT: Recurrent tumor
cc3: Cleaved caspase-3
EdU: 5-Ethynyl-2′-deoxyuridine
FBS: Fetal bovine serum
csFBS: Charcoal-stripped fetal bovine serum
PhR: Phenol red
E2: 17β-Estradiol
*NSG*: NOD SCID gamma
S1P: Sphingosine-1-phosphate
