# Supplementary figures and images for: PAQR8 promotes breast cancer recurrence and confers resistance to multiple therapies
Source: Breast Cancer Res. 2023 Jan 3;25:1. doi: 10.1186/s13058-022-01559-3 (PMC9811758; doi:10.1186/s13058-022-01559-3)

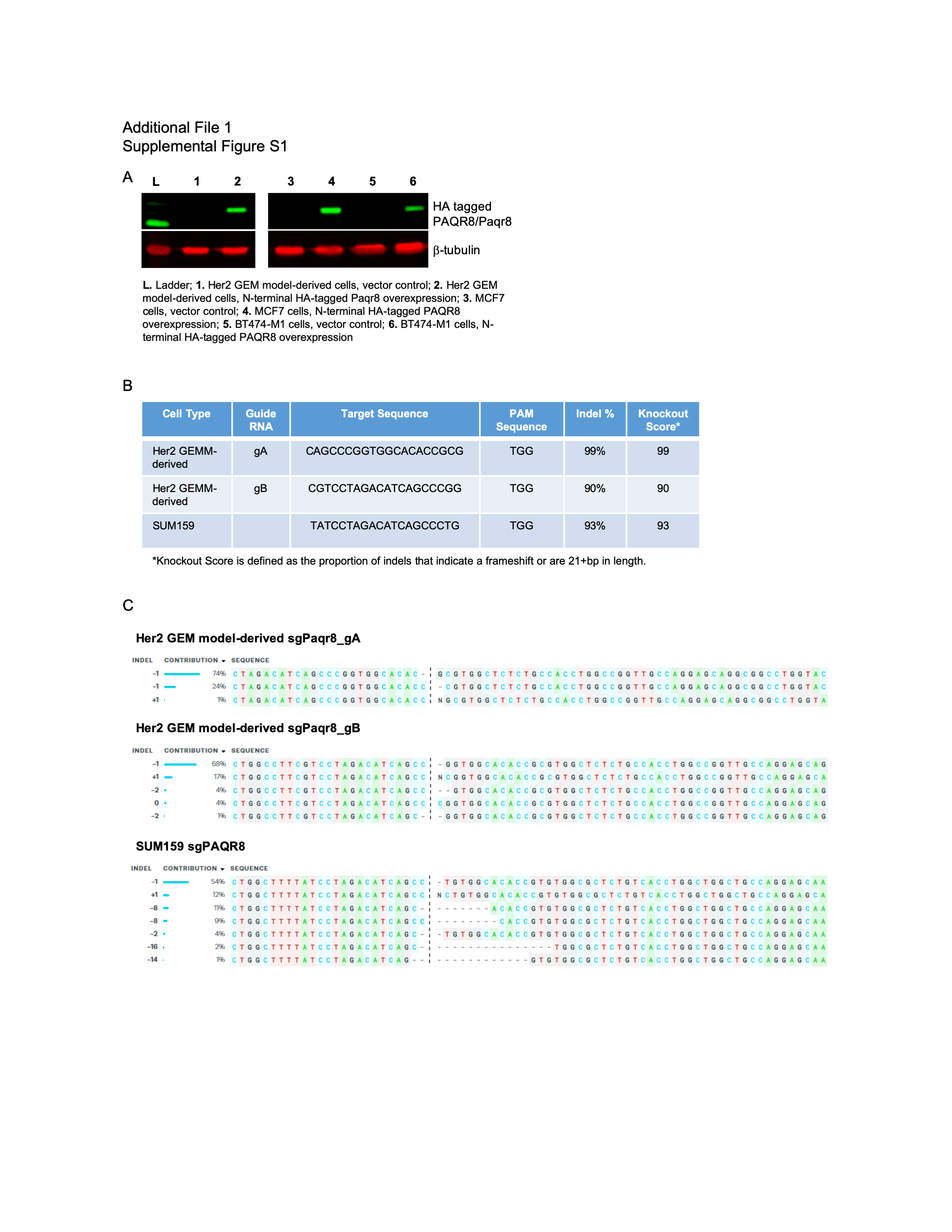

Supplement: Supplementary file 1 — Additional file 1: Figure S1. Validating PAQR8/Paqr8 overexpression and knockout. a Western blot showing anti-HA tagged PAQR8/Paqr8 and anti-β-tubulin loading control. b Synthego ICE analysis results for Paqr8 knockout cells showing guide target sequence, PAM sequence, indel %, and knockout score. c TIDE sequencing results showing relative proportion of specific indels. [file 13058_2022_1559_MOESM1_ESM.tiff]

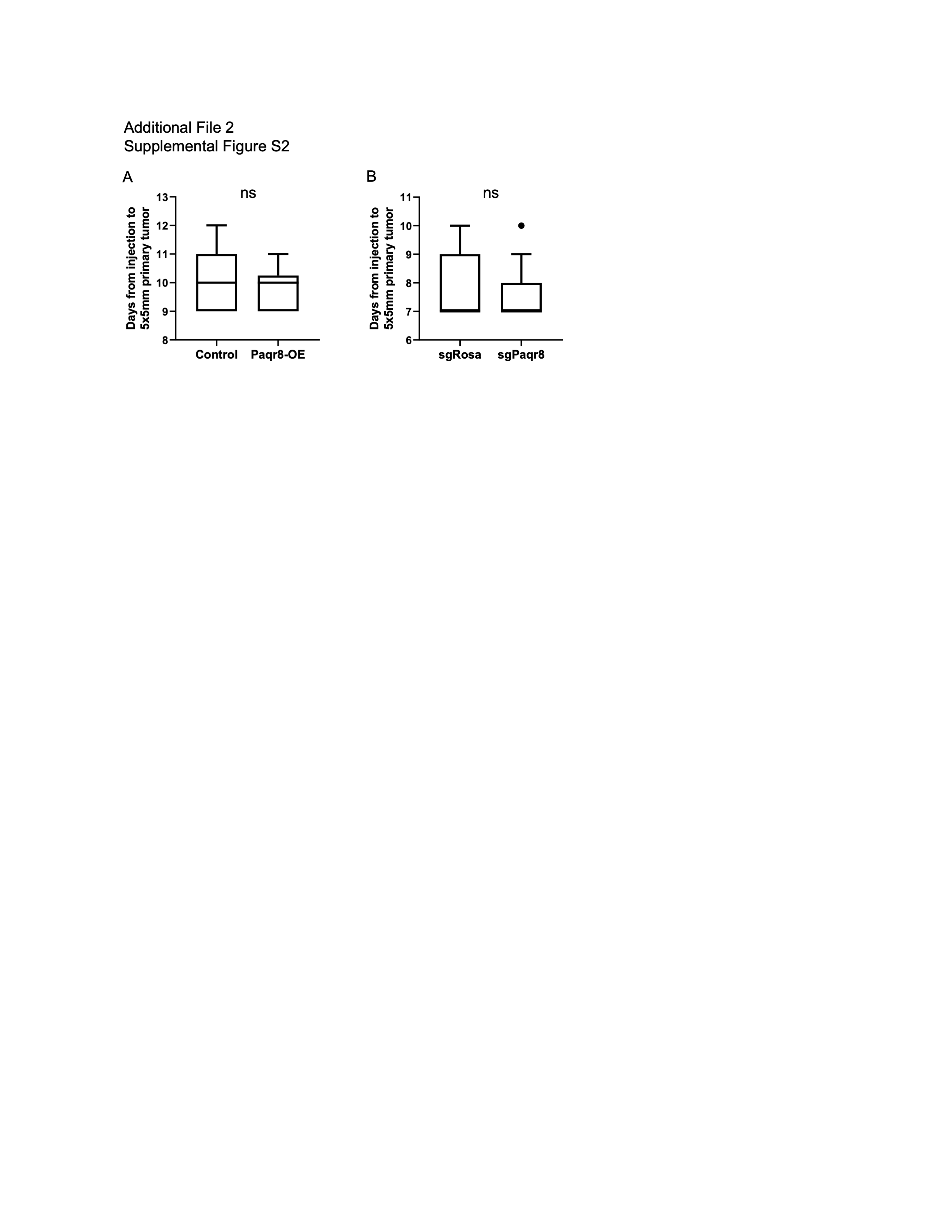

Supplement: Supplementary file 2 — Additional file 2: Paqr8 does not affect the rate of primary tumor growth in the presence of Her2 expression. nu/nu mice were orthotopically injected with Her2-dependent primary mouse cells. Doxycycline in the drinking water maintained Her2 expression during primary tumor formation. Time from injection to palpation of 5x5mm primary tumors is shown. [file 13058_2022_1559_MOESM2_ESM.tiff]

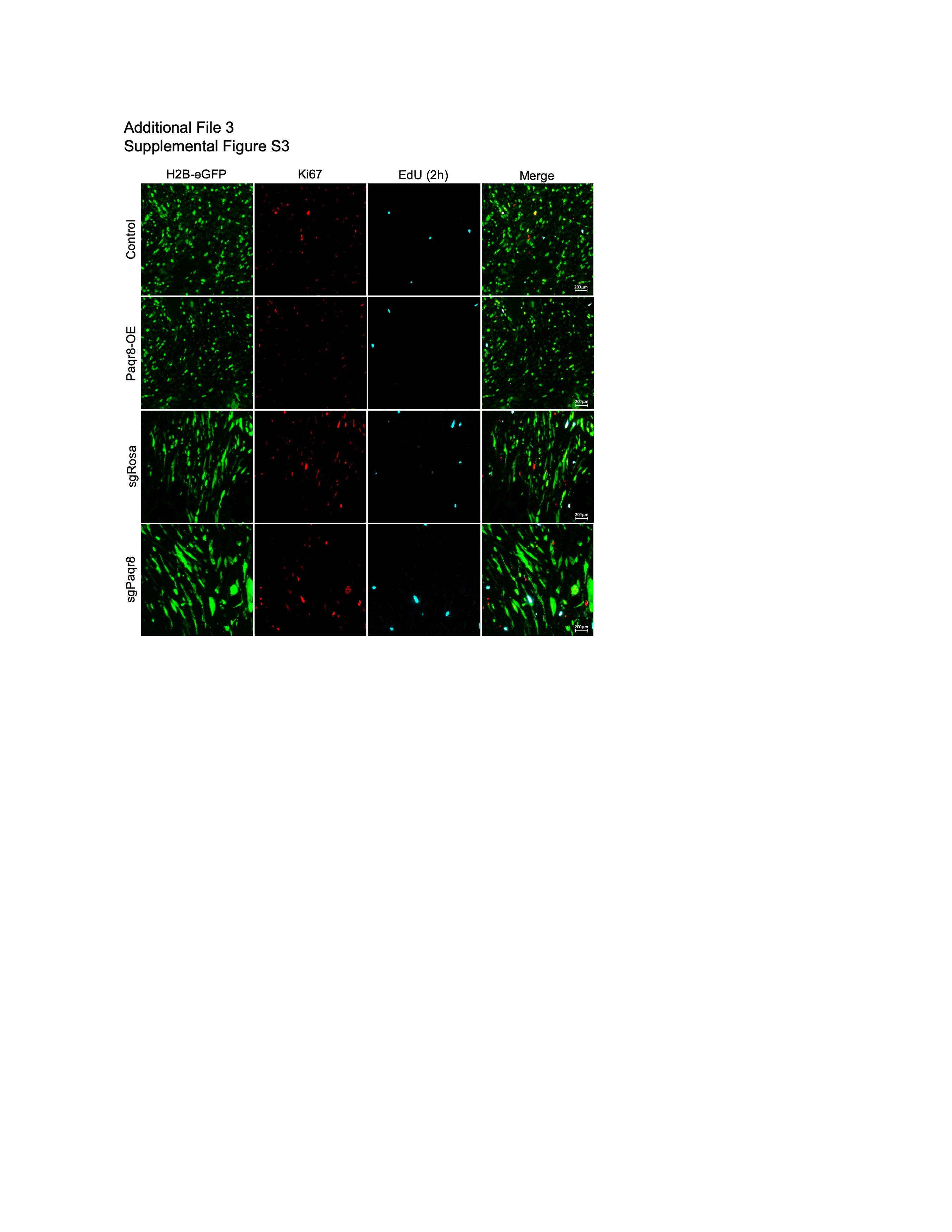

Supplement: Supplementary file 3 — Additional file 3: Paqr8 does not affect cell proliferation following acute Her2 withdrawal in vivo. nu/nu mice harboring Paqr8-OE or Paqr8-KO tumors from orthotopically injected Her2-dependent primary mouse cells three days following Her2 downregulation. Mice were injected with 50mg/kg EdU (i.p.) 2h prior to sacrifice. Residual lesions were harvested, sectioned, and stained by immunofluorescence for EdU and Ki67. Representative images are shown. [file 13058_2022_1559_MOESM3_ESM.tiff]

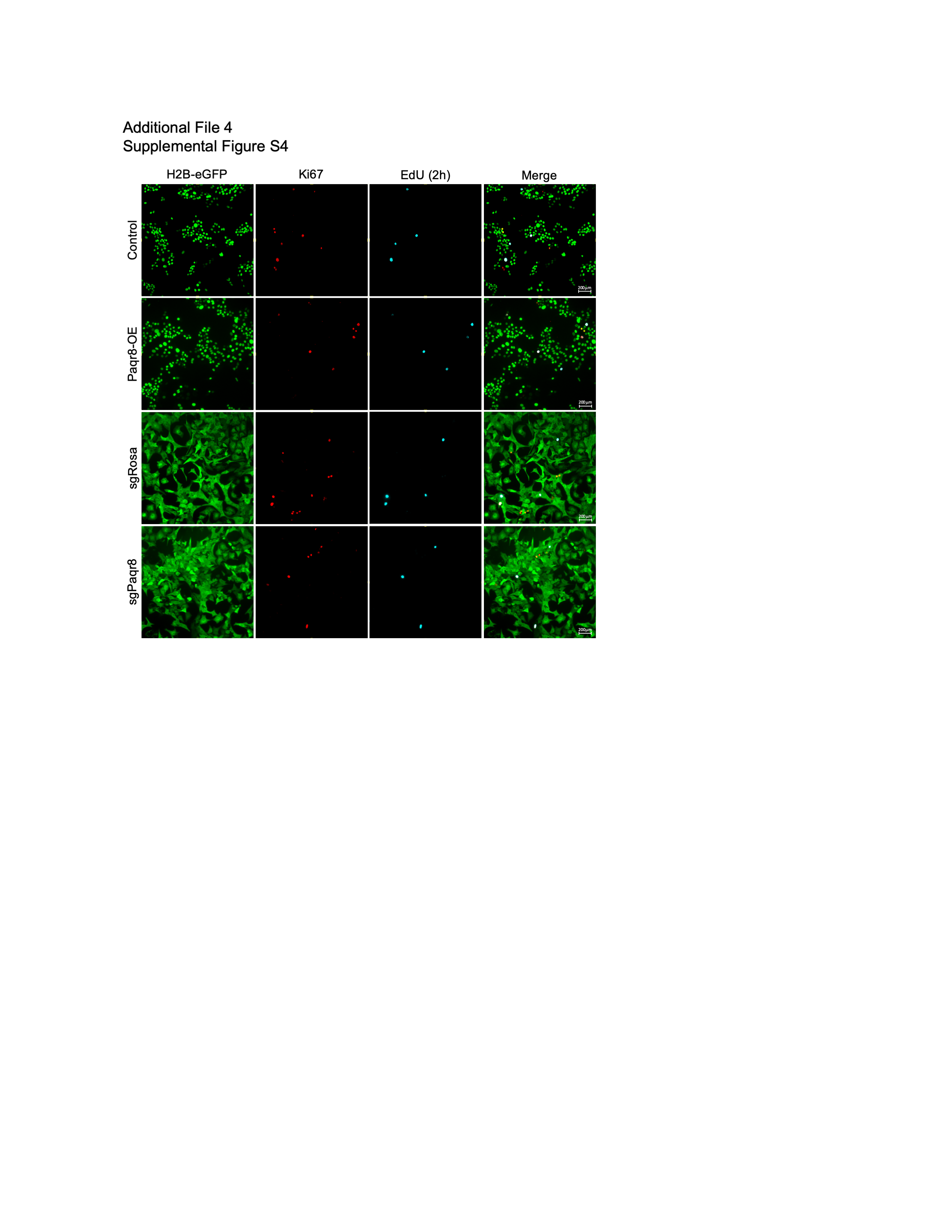

Supplement: Supplementary file 4 — Additional file 4: Paqr8 does not affect proliferation following acute Her2 downregulation in vitro. Her2-dependent primary mouse cells were cultured in 1% serum without doxycycline (Her2 OFF) for 72h and incubated with 10mM EdU for 2h prior to fixation, permeabilization, and staining for EdU and Ki67. Representative images are shown. [file 13058_2022_1559_MOESM4_ESM.tiff]

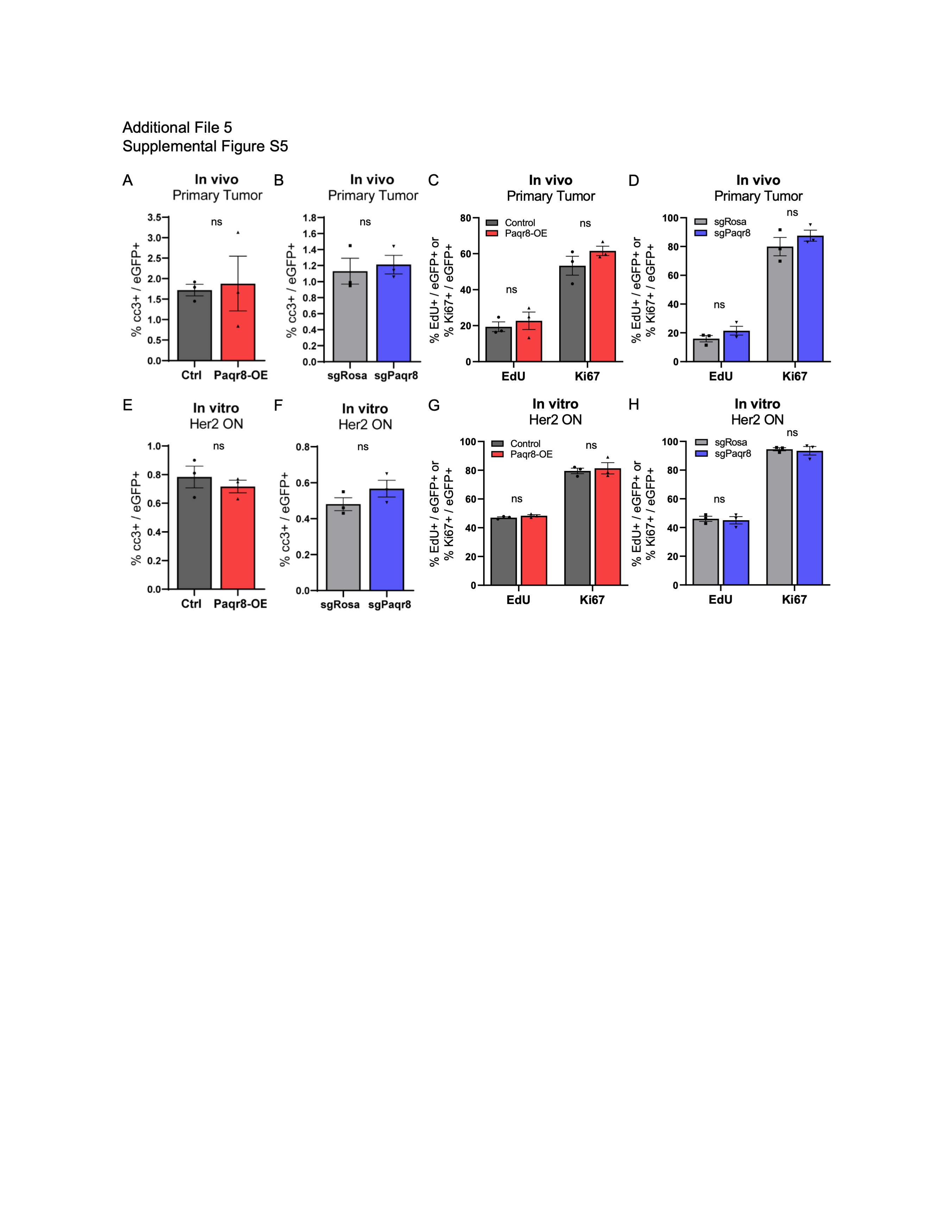

Supplement: Supplementary file 5 — Additional file 5: Paqr8 does not affect rates of apoptosis or cell proliferation in the presence of Her2 expression in vivo or in vitro. nu/nu mice harboring 5x5mm primary tumors from orthotopically injected Her2-dependent Paqr8-OE (a, c) or Paqr8-KO (b, d) primary mouse tumor cells were injected with 50mg/kg EdU (i.p.) 2h prior to sacrifice. Primary tumors were harvested, sectioned, and stained by immunofluorescence for cleaved caspase-3 (cc3), EdU, and Ki67. a-d Percentage of eGFP+ cells that were cc3+, EdU+, or Ki67+ as indicated. Paqr8-OE (e, g) or Paqr8-KO (f, h) Her2-dependent primary cells were cultured in the presence of 10% serum with doxycycline (Her2 ON) and incubated with 10mM EdU for 2h prior to fixation, permeabilization, and staining for cc3, EdU, and Ki67. e-h Percentage of eGFP+ that were cc3+, EdU+, or Ki67+, as indicated. [file 13058_2022_1559_MOESM5_ESM.tiff]

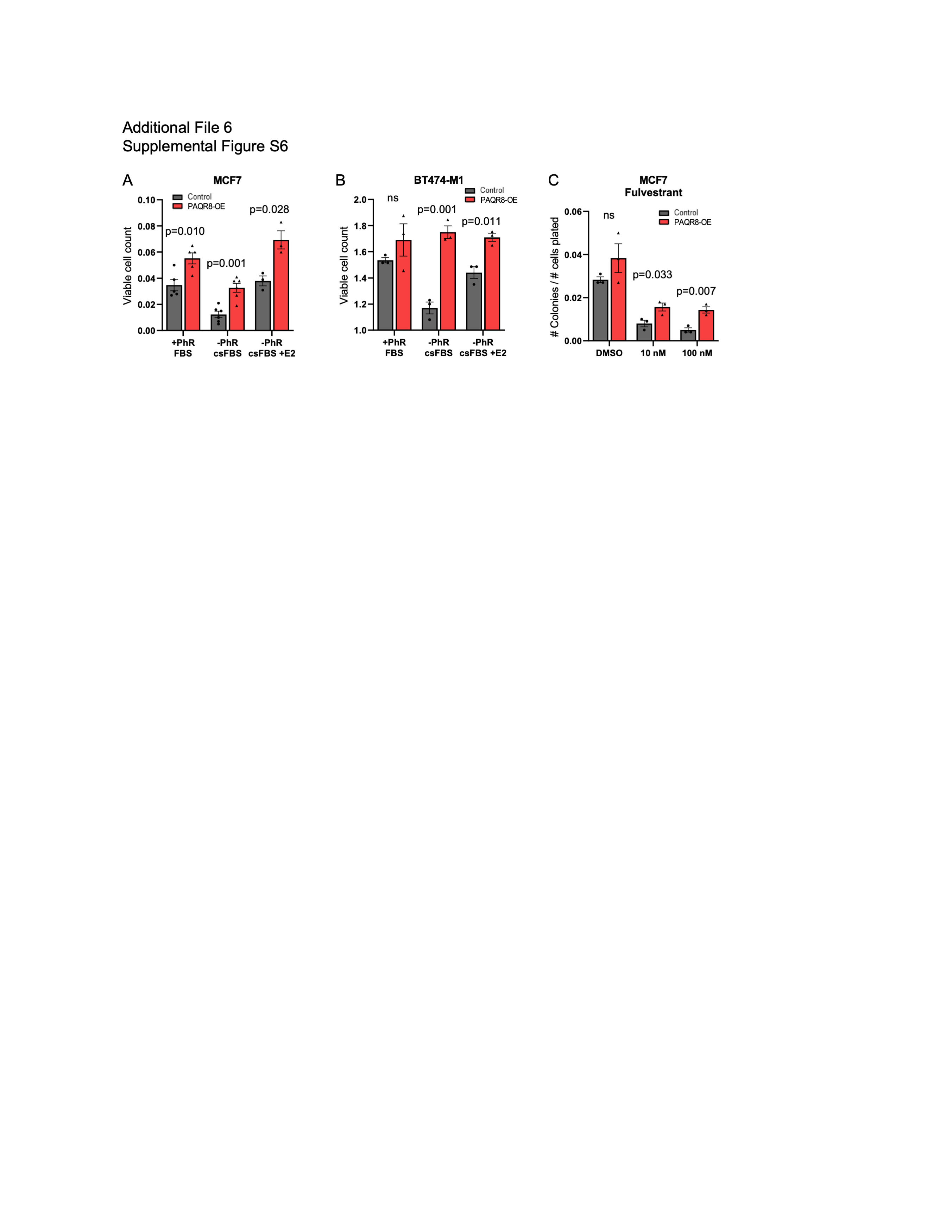

Supplement: Supplementary file 6 — Additional file 6: a Proportion of plated MCF7 cells that formed colonies in +PhR csFBS, -PhR csFBS, or -PhR csFBS +E2 medium. b Viable cell count of BT474-M1 cells in +PhR FBS, -PhR csFBS, or -PhR csFBS +E2 medium. c Proportion of MCF7 cells plated that formed colonies in fulvestrant or vehicle control (DMSO). [file 13058_2022_1559_MOESM6_ESM.tiff]

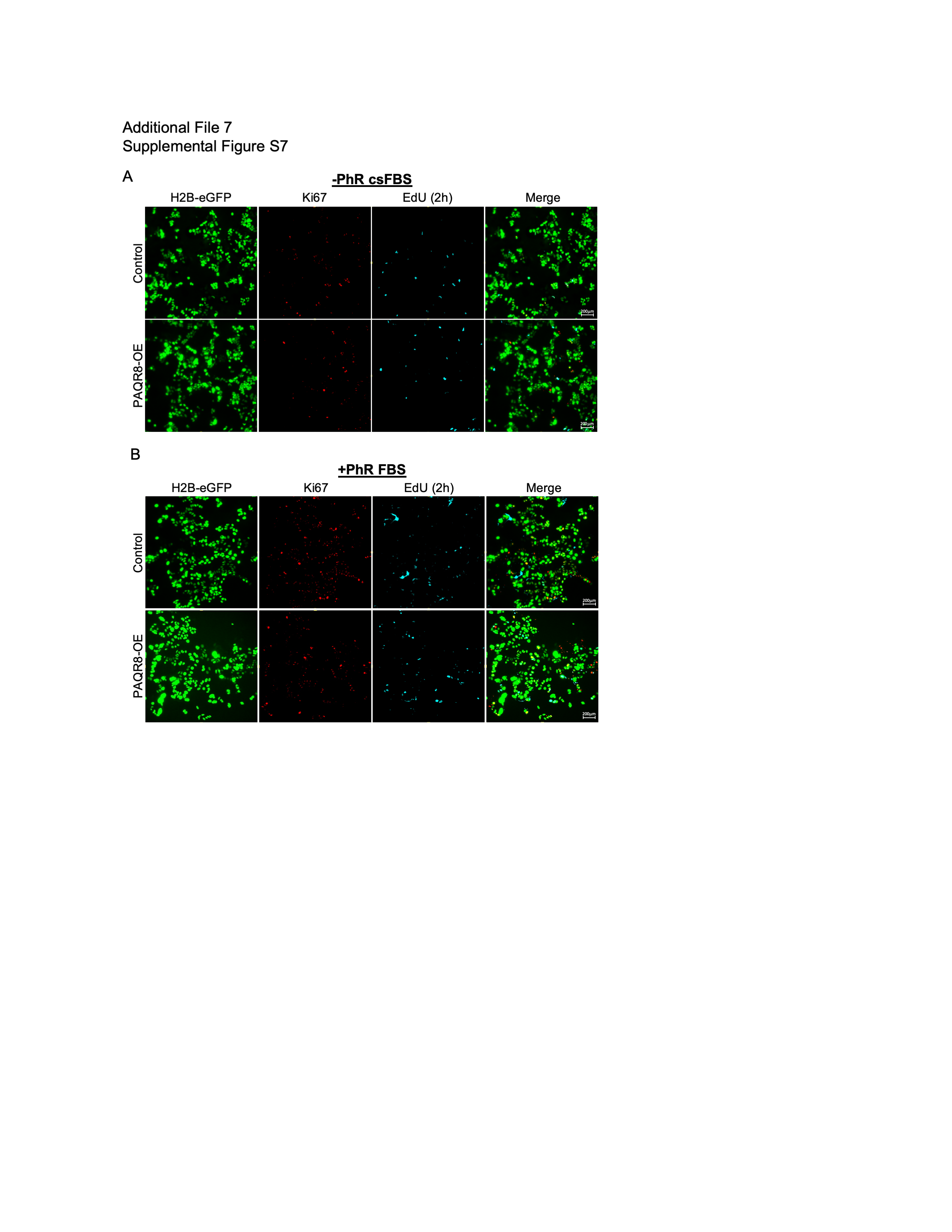

Supplement: Supplementary file 7 — Additional file 7: PAQR8 does not affect proliferation of MCF7 cells following 72h of estrogen deprivation in vitro. MCF7 cells were cultured in either growth medium containing phenol red and fetal bovine serum (+PhR FBS) or estrogen-deprived medium without phenol red containing charcoal-stripped fetal bovine serum (-PhR csFBS) for 72h. Cells were incubated with 10mM EdU for 2h prior to fixation, permeabilization, and immunofluorescence staining for EdU and Ki67. Representative images are shown. [file 13058_2022_1559_MOESM7_ESM.tiff]

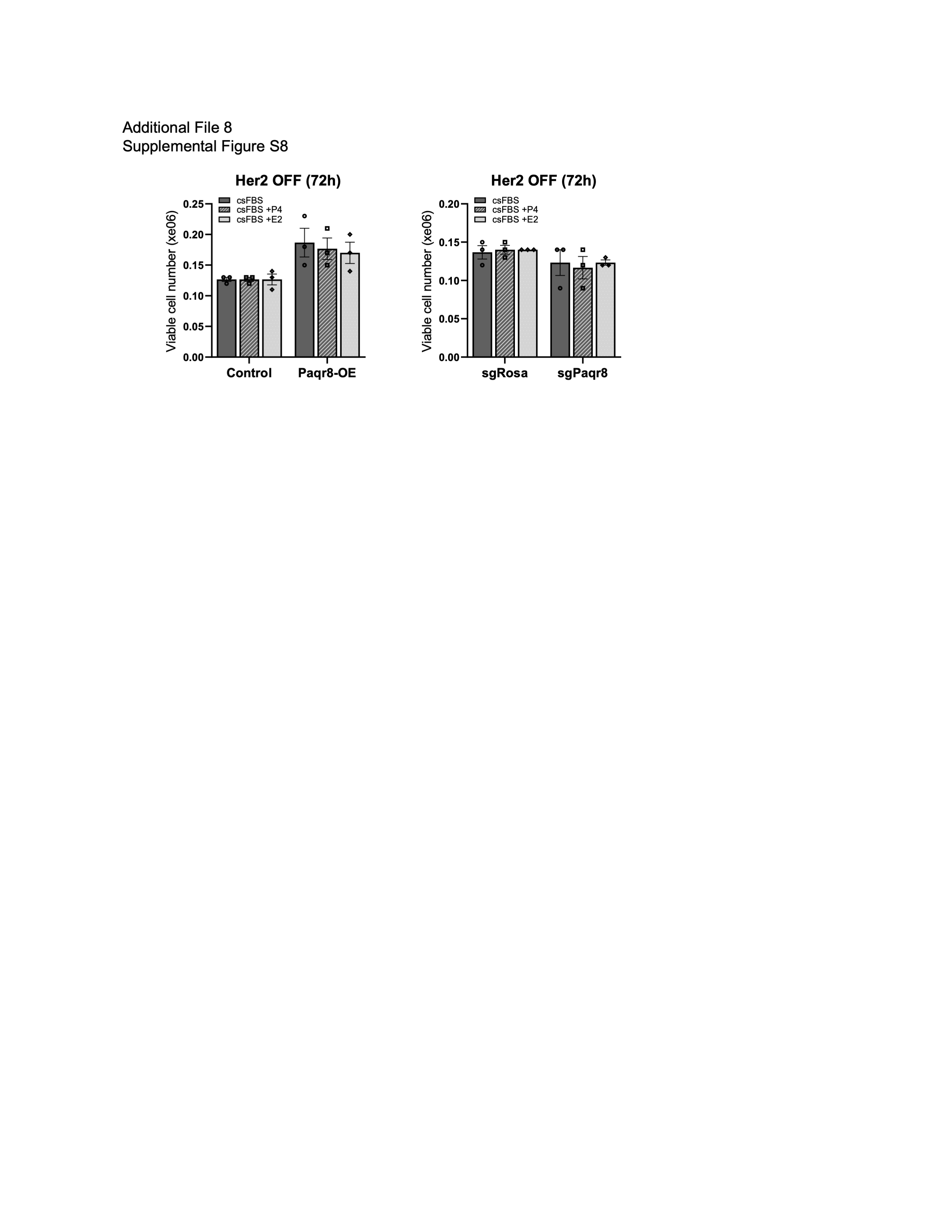

Supplement: Supplementary file 8 — Additional file 8: Effects of Paqr8 on viable cell count following 72h of Her2 withdrawal do not depend on presence of progesterone or estrogen. Her2-dependent primary mouse tumor cells were cultured without doxycycline (Her2 OFF) for 72h. Media contained 1% charcoal-stripped FBS (csFBS), in the presence of 1mM progesterone (P4), 1nM estrogen (E2), or neither. [file 13058_2022_1559_MOESM8_ESM.tiff]

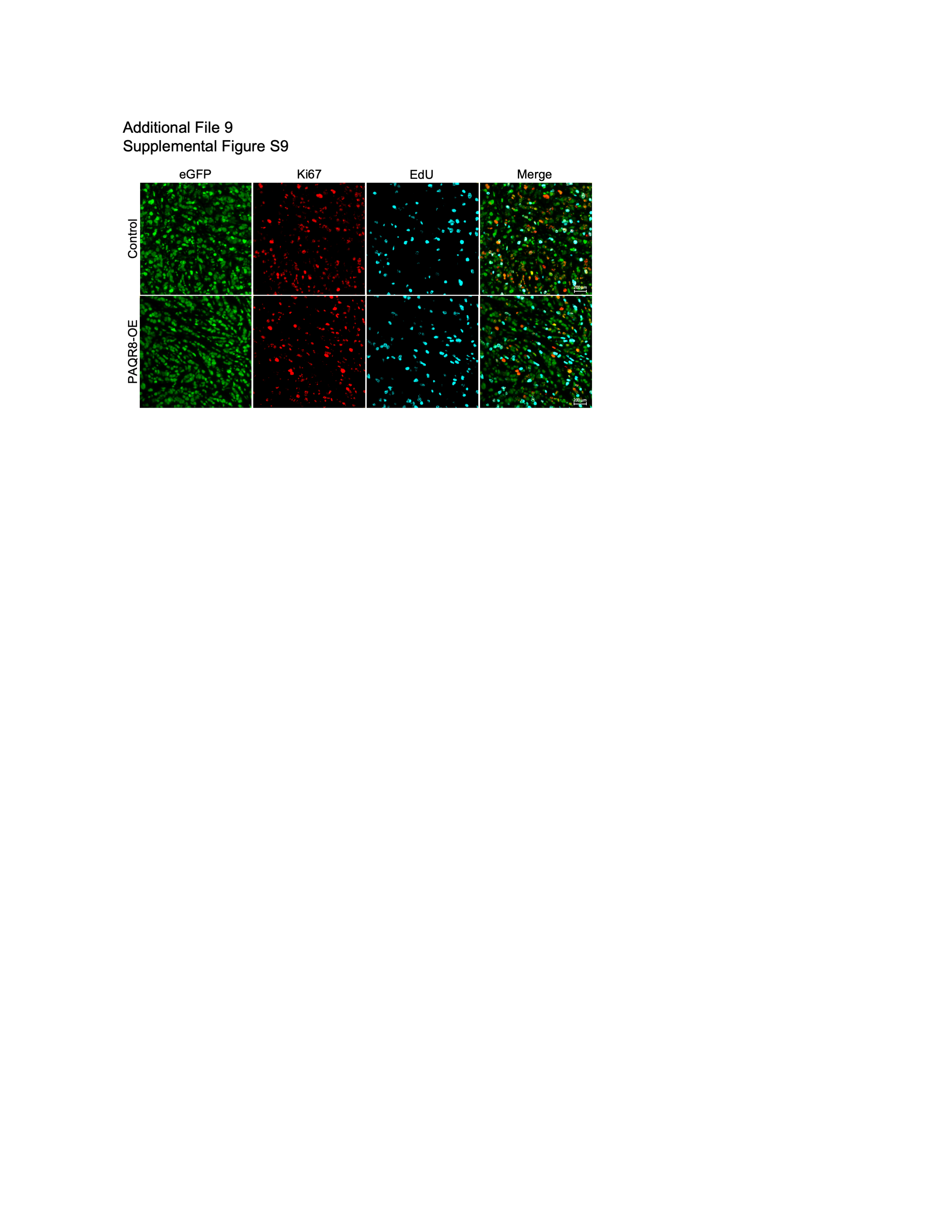

Supplement: Supplementary file 9 — Additional file 9: PAQR8 does not affect proliferation of MCF7 cells in primary tumors formed in NSG mice without estrogen supplementation. NSG mice harboring orthotopic MCF7 tumors were injected with 50mg/kg of EdU (i.p.) 2h prior to sacrifice. Tumors were harvested and fixed in 4% paraformaldehyde, paraffin embedded, sectioned and stained by immunofluorescence for EdU and Ki67. Representative images are shown. [file 13058_2022_1559_MOESM9_ESM.tiff]

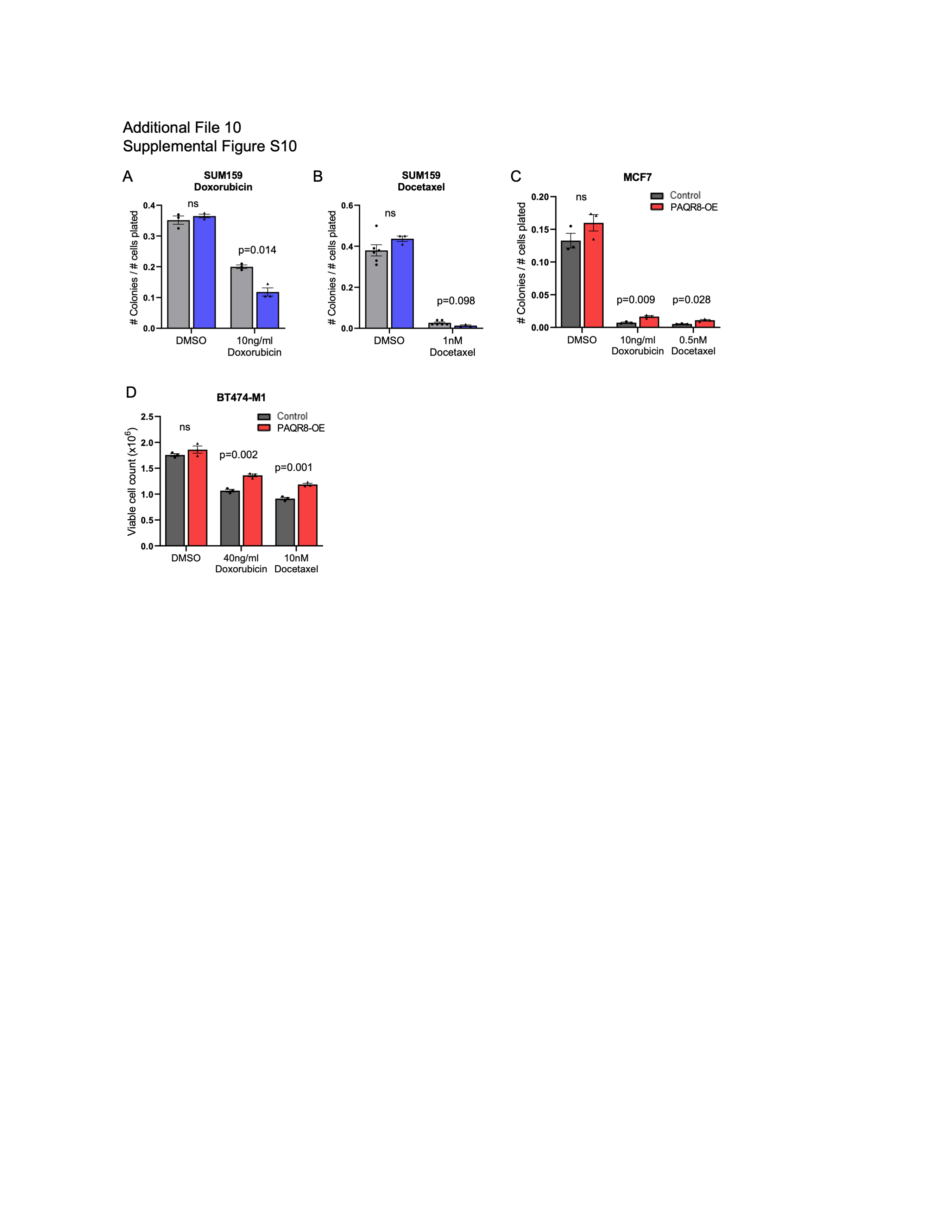

Supplement: Supplementary file 10 — Additional file 10: a Proportion of plated SUM159 cells that formed colonies in the presence of doxorubicin or vehicle control (DMSO). b Proportion of plated SUM159 cells that formed colonies in the presence of docetaxel or vehicle control (DMSO). c Proportion of plated MCF7 cells that formed colonies in the presence of doxorubicin, docetaxel, or vehicle control (DMSO). d Viable cell counts of BT474-M1 cells in the presence of doxorubicin, docetaxel, or vehicle control (DMSO). [file 13058_2022_1559_MOESM10_ESM.tiff]

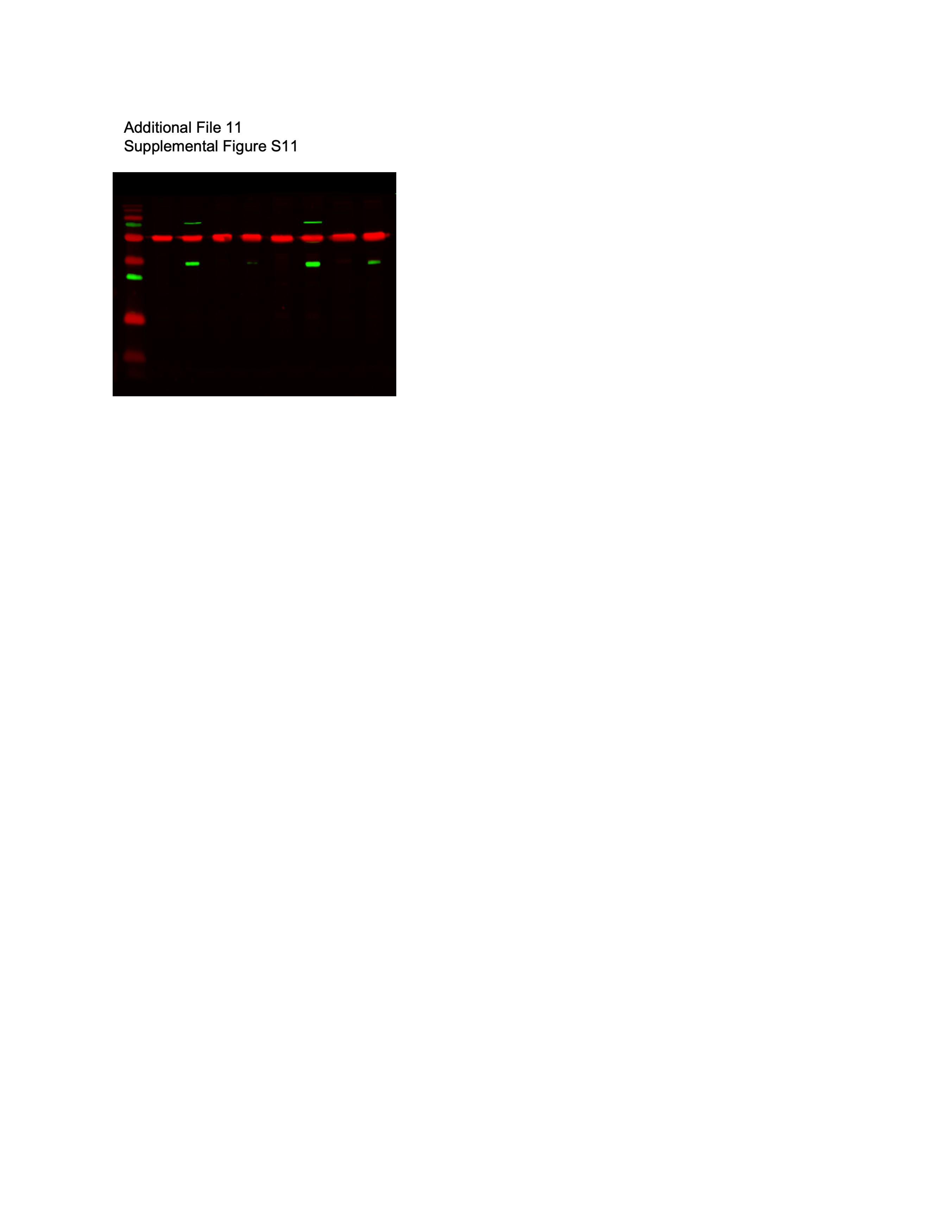

Supplement: Supplementary file 11 — Additional file 11: Full western blot image corresponding to Figure S1a. [file 13058_2022_1559_MOESM11_ESM.tiff]
